# Limited antigenic diversity of *Plasmodium falciparum* apical membrane antigen 1 supports the development of effective multi-allele vaccines

**DOI:** 10.1186/s12916-014-0183-5

**Published:** 2014-10-16

**Authors:** Ulrich Terheggen, Damien R Drew, Anthony N Hodder, Nadia J Cross, Cleopatra K Mugyenyi, Alyssa E Barry, Robin F Anders, Sheetij Dutta, Faith HA Osier, Salenna R Elliott, Nicolas Senn, Danielle I Stanisic, Kevin Marsh, Peter M Siba, Ivo Mueller, Jack S Richards, James G Beeson

**Affiliations:** The Burnet Institute of Medical Research and Public Health, 85 Commercial Road, Melbourne, Victoria 3004 Australia; Department of Medicine, University of Melbourne, Melbourne, Victoria Australia; Walter and Eliza Hall Institute, Melbourne, Australia; Centre for Geographic Medicine, Coast, Kenya Medical Research Institute, Kilifi, Kenya; Department of Medical Biology, University of Melbourne, Melbourne, Victoria Australia; La Trobe University, Melbourne, Australia; Walter Reed Army Institute, Silver Spring, MD USA; Papua New Guinea Institute of Medical Research, Madang, Papua New Guinea; Swiss Tropical and Public Health Institute, Basel, Switzerland; Department of Microbiology, Monash University, Clayton, Victoria Australia

**Keywords:** Malaria, *Plasmodium falciparum*, Vaccines, Immunity, Apical membrane antigen 1, Cross-reactive antibodies

## Abstract

**Background:**

Polymorphism in antigens is a common mechanism for immune evasion used by many important pathogens, and presents major challenges in vaccine development. In malaria, many key immune targets and vaccine candidates show substantial polymorphism. However, knowledge on antigenic diversity of key antigens, the impact of polymorphism on potential vaccine escape, and how sequence polymorphism relates to antigenic differences is very limited, yet crucial for vaccine development. *Plasmodium falciparum* apical membrane antigen 1 (AMA1) is an important target of naturally-acquired antibodies in malaria immunity and a leading vaccine candidate. However, AMA1 has extensive allelic diversity with more than 60 polymorphic amino acid residues and more than 200 haplotypes in a single population. Therefore, AMA1 serves as an excellent model to assess antigenic diversity in malaria vaccine antigens and the feasibility of multi-allele vaccine approaches. While most previous research has focused on sequence diversity and antibody responses in laboratory animals, little has been done on the cross-reactivity of human antibodies.

**Methods:**

We aimed to determine the extent of antigenic diversity of AMA1, defined by reactivity with human antibodies, and to aid the identification of specific alleles for potential inclusion in a multi-allele vaccine. We developed an approach using a multiple-antigen-competition enzyme-linked immunosorbent assay (ELISA) to examine cross-reactivity of naturally-acquired antibodies in Papua New Guinea and Kenya, and related this to differences in AMA1 sequence.

**Results:**

We found that adults had greater cross-reactivity of antibodies than children, although the patterns of cross-reactivity to alleles were the same. Patterns of antibody cross-reactivity were very similar between populations (Papua New Guinea and Kenya), and over time. Further, our results show that antigenic diversity of AMA1 alleles is surprisingly restricted, despite extensive sequence polymorphism. Our findings suggest that a combination of three different alleles, if selected appropriately, may be sufficient to cover the majority of antigenic diversity in polymorphic AMA1 antigens. Antigenic properties were not strongly related to existing haplotype groupings based on sequence analysis.

**Conclusions:**

Antigenic diversity of AMA1 is limited and a vaccine including a small number of alleles might be sufficient for coverage against naturally-circulating strains, supporting a multi-allele approach for developing polymorphic antigens as malaria vaccines.

**Electronic supplementary material:**

The online version of this article (doi:10.1186/s12916-014-0183-5) contains supplementary material, which is available to authorized users.

## Background

Malaria continues to have a profound impact on the health of children and adults around the world, causing approximately 219 million clinical cases and 660,000 deaths per year [1]. Even though the scale-up of malaria-control interventions has resulted in a notable reduction in morbidity and mortality over the last decade, drug resistance remains a significant concern, and a cost-effective vaccine could play a major role in control and eventual elimination of the disease.

Understanding the mechanisms by which naturally-acquired immunity to malaria protects against death and severe disease is important for informing the rational design and development of effective vaccines. Antibodies constitute a major component of naturally-acquired immunity [2-4]. The merozoite form of the parasite, which invades red blood cells, expresses antigens that are prominent antibody targets [5]. Merozoite antigens are attractive vaccine targets because antibodies to these antigens inhibit red blood cell invasion and promote opsonic phagocytosis and antibody-dependent cellular inhibition that limit blood stage replication and prevent disease [6-9]. Furthermore, antibodies to merozoite antigens are associated with protection from malaria [10], and several merozoite antigen-based vaccines have shown protective efficacy in animal models [6,11]. A major challenge in developing merozoite-based vaccines, and other vaccines based on antigens that are targets of natural immunity, is overcoming potential antigenic diversity. Most major immune targets, and many vaccine candidates, show substantial polymorphism in sequence that have evolved to facilitate immune evasion. Vaccine approaches are needed to account for this polymorphism such that they will cover the majority of strains causing infection and disease. Although sequence polymorphism has been described for many antigens, knowledge is very limited on the extent of antigenic diversity (defined by antigen reactivity to human antibodies) and how polymorphisms relate to antigenic diversity for most leading candidate antigens, yet this is crucial for advancing vaccine development.

There are more than 40 different merozoite antigens on the surface or in the apical organelles of merozoites, few of which have been investigated as immune targets [5,6,10]. One important target, and leading vaccine candidate, is apical membrane antigen 1 (AMA1), which plays an essential role in erythrocyte invasion [12]. Antibodies to AMA1 are highly prevalent in malaria-exposed individuals and their prevalence increases with age as naturally-acquired immunity develops [13]. Antibodies to AMA1 have been associated with reduced risk of clinical malaria in prospective studies [14-16] and *in vitro* data indicate that AMA1 antibodies can inhibit parasite invasion of erythrocytes [17-19]. AMA1 is a promising blood stage vaccine candidate which is presently being tested in clinical trials. A recent phase II trial of a monovalent AMA 1 vaccine in one- to six-year-old children in Mali showed 65% strain specific efficacy [20]. However, AMA1 is a highly polymorphic protein with more than 60 polymorphic sites and more than 200 haplotypes per population [21,22], one of the most polymorphic of all merozoite antigens. Immunization with one allele of AMA1 may not protect against parasites expressing different AMA 1 alleles, as highlighted by the Mali trial; there was no overall protection against clinical malaria, but there was evidence of protection against malaria caused by vaccine-like alleles [20]. While sequence analysis has been used to classify AMA1 alleles into related groups that might show cross-reactive immunity, the antigenic diversity of AMA1 and cross-reactivity of antibodies are poorly understood, and it is unclear how sequence polymorphisms and sequence-based groupings relate to antigenic diversity and escape from acquired human antibodies. There are only limited data on antigenic diversity in human studies and limited data to understand how sequence diversity is related to antigenic diversity, which is an impediment to vaccine development. Understanding these issues is essential for advancing AMA1-based vaccines. AMA1 also serves as an ideal model to examine antigenic diversity more broadly, the significance of polymorphism in vaccine development and the feasibility of developing multi-allele vaccines based on polymorphic antigens.

We sought to define antigenic diversity of AMA1 and use this knowledge to understand which AMA1 alleles could be included in a multi-allele vaccine to achieve the broadest coverage of AMA1 diversity, and establish principles that could be applied to other polymorphic vaccine antigens. Antibody reactivity to various geographically-diverse AMA1 alleles was examined among children of different ages and adults from two geographically diverse malaria endemic regions (Papua New Guinea and Kenya). We examined the relationship between antigenic diversity and sequence diversity, and sought to establish whether overall antigenic diversity of AMA1 is limited and might ultimately be reduced to a small number of major serotypes. To achieve these objectives, we developed a novel approach that we named multiple antigen competition enzyme-linked immunosorbent assay (ELISA) (MACE) that can also be used to define antigenic diversity of other polymorphic antigens.

## Methods

### Study cohorts and sample collection

Serum samples were collected from a cross-sectional study in Madang Province, Papua New Guinea (PNG) in 2007, and included 118 individuals: 49 adults (median age 28 years) and 69 children (median age 6 years). From these samples we prepared pools of AMA1 antibody positive samples for testing in competition ELISAs; one pool was made from children’s samples (n = 31; median age 7 years (range 4 to 10)) and one from adult samples (n = 42; median age 28 years (range 16 to 53)). To prepare the pools, all sera were first tested in standard ELISA for immunoglobulin G (IgG) reactivity against five different recombinant AMA1 alleles (3D7, W2mef, FVO, 7G8, and HB3). After screening, we excluded antibody negative samples and samples with low antibody reactivity (defined as below the 25^th^ centile). All samples included in the pools were antibody positive to all five AMA1 alleles, and antibody reactivity to different alleles was highly correlated (>0.9 for all comparisons), as we have found previously in this population [16]. In preparing pools, an equal volume of all individual samples was used, and substantial numbers of samples were included in pools to account for variation in antibody levels and cross-reactivity among individual samples.

Samples were also obtained from an extended longitudinal study in Kilifi, Kenya (Ngerenya cohort). More than 300 children were included in the original Kenyan cohort [23]; in our study we examined serum from a subset of 42 children who were positive for AMA1 antibody responses and were present for screening and sample collection at two different time points, October 2002 and October 2004. From these samples we prepared a pool of AMA1 antibody positive samples for testing in competition ELISAs using samples collected from the same children at the two different times. To select samples for inclusion in the pools, individual samples were first screened for IgG reactivity to three AMA1 alleles (3D7, W2mef and HB3) in standard ELISA. The levels and proportion of positive samples were lower than for the PNG samples. For preparation of pools we selected the top quartile of responders to AMA1-3D7; however, antibodies to different AMA1 alleles were highly correlated (correlation co-efficient 0.77 to 0.95) and all individuals had antibody reactivity greater than the group median for all AMA1 alleles. The median age (range) of children in the sample pools was 6.5 years (2.1 to 7.6) for October 2002, and 8.3 years (4.2 to 9.8) for October 2004. Sera from unexposed Australian blood donors donated by the Red Cross Blood Bank were used as negative controls in ELISAs and antibody positive samples were defined as those with reactivity greater than the median + three standard deviations (SD) of the Australian controls. Ethical approval was granted by the Kenya National Research Ethics Committee, the Medical Research Advisory Council PNG, the Walter and Eliza Hall Institute, and Alfred Hospital Human Research and Ethics Committees. Written informed consent was obtained from all participants or their guardians.

### AMA1 alleles

Eleven alleles of the *Plasmodium falciparum* AMA1 antigen were used in this study (3D7, D10, W2mef, 7G8, FVO, HB3, XIE, Pf2004, Pf2006, M24, 102-1). The origins of *P. falciparum* isolates expressing the selected AMA1 alleles are listed in Table 1. Alleles were chosen to represent the broad genetic diversity of AMA1 [24], based on published *P. falciparum* AMA1 sequences (available from the Protein Data Bank). Sequence alignment of the 11 alleles identified 52 polymorphic amino acid (aa) positions: 7 in the prodomain, 28 in DI, 8 in DII and 9 in DIII [see Additional file 1: Figure S1]. The number of sequence differences between any two alleles ranged from 8 to 27 (see Additional file 1: Figure S2).

**Table 1:** ***P. falciparum*** **AMA1 alleles selected for this study**

**Isolate**	**Origin**	**Reference**
**3D7**	Amsterdam Airport, origin unknown	[25]
**D10**	Papua New Guinea	[26]
**W2Mef**	Southeast Asia	[27]
**7G8**	Peru	[28]
**FVO**	Vietnam	[29]
**HB3**	Honduras	[30]
**XIE**	PNG	[31]
**Pf2004**	Ghana	[32]
**Pf2006**	Ghana	[32]
**M24**	Kenya	[33,34]
**102-1**	Sudan	[35]

AMA1, apical membrane antigen 1.

### Preparation of recombinant AMA1

Recombinant AMA1 proteins used in this study were expressed, purified and refolded using established protocols [19,24,36]. Nucleotide sequences were amplified from genomic DNA using Pfu DNA polymerase and oligonucleotide primers. The amplified products were digested with BamHI and Xho1, and ligated into pProEXHT-B 6xHis, and transformed into *Escherich****i****a coli* strain BL21. (Proteins were solubilized in 6 M guanidine-HCL, which completely denatures the recombinant proteins). After purification on nickel resin, AMA1 protein was refolded with reduced and oxidized glutathione redox pairs. Refolded AMA1 was further purified by anion exchange chromatography, followed by reversed-phase, high performance liquid chromatography (RP-HPLC). Refolded AMA1 was identified by a shift in the monomer peak on RP-HPLC and a migration shift on SDS-PAGE when compared to a reduced sample of the refolded AMA1 preparation. Full details of preparation can be found elsewhere [19].

### Competition ELISAs

Standard ELISAs to measure IgG to recombinant AMA1 were performed using established methods. Serum samples were tested in single and multiple antigen competition ELISA (MACE) against 11 AMA1 alleles. A novel method, MACE, was developed by modifying the conventional competition ELISA assay to allow cross-reactivity among several alleles of one protein to be examined. In a conventional competition ELISA assay, human antibodies are pre-incubated with a single competing antigen before adding to wells coated with a different allelic variant of the same antigen and proceeding as for a standard ELISA. In MACE, human antibodies are pre-incubated with combinations of up to four competing heterologous recombinant alleles. A total of 75 different combinations were used for competition. The level of inhibition in the presence of one or more competitor alleles indicates the degree of cross-reactivity with the tested allele used to coat the wells [see Additional file 1: Figure S4].

ELISAs were carried out using 96-well polystyrene Maxisorp plates (Nunc, Roskilde, Denmark) coated with one of eleven recombinant AMA-1 proteins at a concentration of 0.5 μg/ml in phosphate-buffered saline (PBS) and were incubated overnight at 4°C. Plates were blocked using 0.1% casein in PBS-tween 0.05%. Plasma was diluted to a concentration of 1:1,000 and one or combinations of several competing recombinant proteins (AMA-1 variants) were added in excess, each at 5 μg/ml. Prior to performing competition ELISAs, we optimized the conditions of our assays to ensure that antibody reactivity with pools was below the saturation point and the serum concentration gave an Optical Density (OD) reading at 405 nm that was in the linear part of a titration curve showing the relationship between antibody concentration and OD (for example, see [37]). Optimization included titration of serum dilutions and antigen coating concentration, and optimization of secondary antibody concentrations and incubation times for the substrate. Additionally, we titrated the concentration of the competitor antigen to determine the concentration required to fully saturate antibody binding and achieve maximal inhibition. A concentration 5 μg/ml of competition antigen was well above the saturation threshold and was used in all competition ELISAs [see Additional file 1: Figure S3). For each of the 11 AMA-1 variants used as coating antigen, the homologous allele and the heterologous recombinant AMA-1 antigen combinations were added as competitor. The homologous competitor served as internal control for competition [see Additional file 1: Figure S4). The plasma/competitor mix was allowed to pre-incubate at room temperature for 30 minutes and then added to the plate (following washing) for 2 hours. Incubations were done using 50 μl volume per well in duplicate and washes between each incubation were carried out using PBS-tween 0.05%. All incubations occurred at room temperature. To determine total IgG to plate-bound antibodies, horseradish peroxide (HRP)-conjugated sheep anti-human IgG (Millipore, North Ryde, Australia) at 1 in 5,000 was used and allowed to incubate for one hour. ABTS (2,2′-azinobis(3-ethylbenzthiazolinesulfonic acid)) substrate (Sigma-Aldrich, Castle Hill, Australia) was added to develop color and the reaction was stopped using 1% sodium dodecyl sulfate (SDS) after 20 minutes in the dark. The optical density of each sample was measured at 405 nm (ABTS) using a plate reader (Thermo Fisher Scientific, Scoresby, Victoria, Australia). The mean of duplicate wells was calculated and background (wells coated with antigen and incubated with PBS plus competitor) was subtracted for each sample (using ScanIt MulticanPro software). Results on plates were standardized to adjust for plate-to-plate variance using positive control pools on each plate tested [16], and the mean plus three standard deviations of the OD for Australian negative control sera was used as the cut-off for seropositivity.

### Data analysis

Cross-reactivity was defined as degree of inhibition of antibody reactivity by one or more competitor. Standardized OD values were used for calculating the proportion of AMA1 specific reactivity to the coating AMA1 allele that was competed by the heterologous competitor(s) ((heterologous competition – homologous competition)/(no competition – homologous competition) × 100) [see Additional file 1: Figure S4]. ClustalWeb 2.1 was used for amino acid sequence alignments. Statistical analysis was performed using GraphPad Prism 5 (Graphpad Software). Correlations between cross-reactivity and sequence polymorphisms between AMA1 alleles were calculated using Spearman’s rank correlation coefficient (ρ). Two-tailed *P* values were calculated for each rho to determine the significance of correlation. Wilcoxon’s rank sum test was used to compare the level of antibody cross-reactivity between groups, except for comparisons of Kenyan children at two time points for which the Wilcoxon’s signed rank sum test was used.

To investigate phylogenetic relationships among AMA1 sequences, a network analysis was performed for 873 AMA1 ectodomain sequences (residues 148 to 553) available from GenBank including the 11 reference alleles used in the present study. A second network analysis was performed restricted to sequences available from our study populations: this included 49 sequences from Chonyi, Kilifi District, Kenya and 31 from Mugil, Madang Province, PNG. Network analysis was performed using Phylogenetic Network software version 4.6.1.1 together with the add-ons DNA Alignment *and* Network Publisher (Fluxus-engineering, Germany). The network analysis was based on the Median Joining algorithm which connects haplotypes on the basis of the numbers of shared alleles and allows for the multiple connections between haplotypes arising through recombination. Gene accession numbers: KF698984 to KF699059 and FN869649 to FN869697.

## Results

### Antibody cross-reactivity to different AMA1 alleles

Competition ELISAs with human antibodies were used to examine antigenic diversity of AMA1 and investigate whether strain-specificity of human antibody responses to AMA1 could potentially be overcome with the inclusion of a limited number of alleles in a multi-allele vaccine. Competition ELISAs were required because measuring antibodies to different AMA1 alleles by standard ELISA does not sufficiently discriminate the levels of cross-reactivity and allele-specificity of antibodies. AMA1 alleles of diverse geographical origins were selected (Table 1). Phylogenetic tree analysis has shown previously that these alleles broadly represent global AMA1 diversity [24]. In order to efficiently test the many antigen competition comparisons in this study, we prepared a pool of serum samples from PNG children (n = 31; median age 7 years) and a pool of adult samples (n = 42; median age 28 years) selecting those who were antibody positive after initial screening for reactivity to AMA1 in standard ELISA. We confirmed the use of the pools as an appropriate strategy by demonstrating that competition ELISA results using pools revealed very similar patterns and levels of antibody cross-reactivity as those obtained when testing all samples individually [see Additional file 1: Figure S5]. We have previously reported the use of pools to measure other anti-malarial antibodies [38].

**Figure 1:**
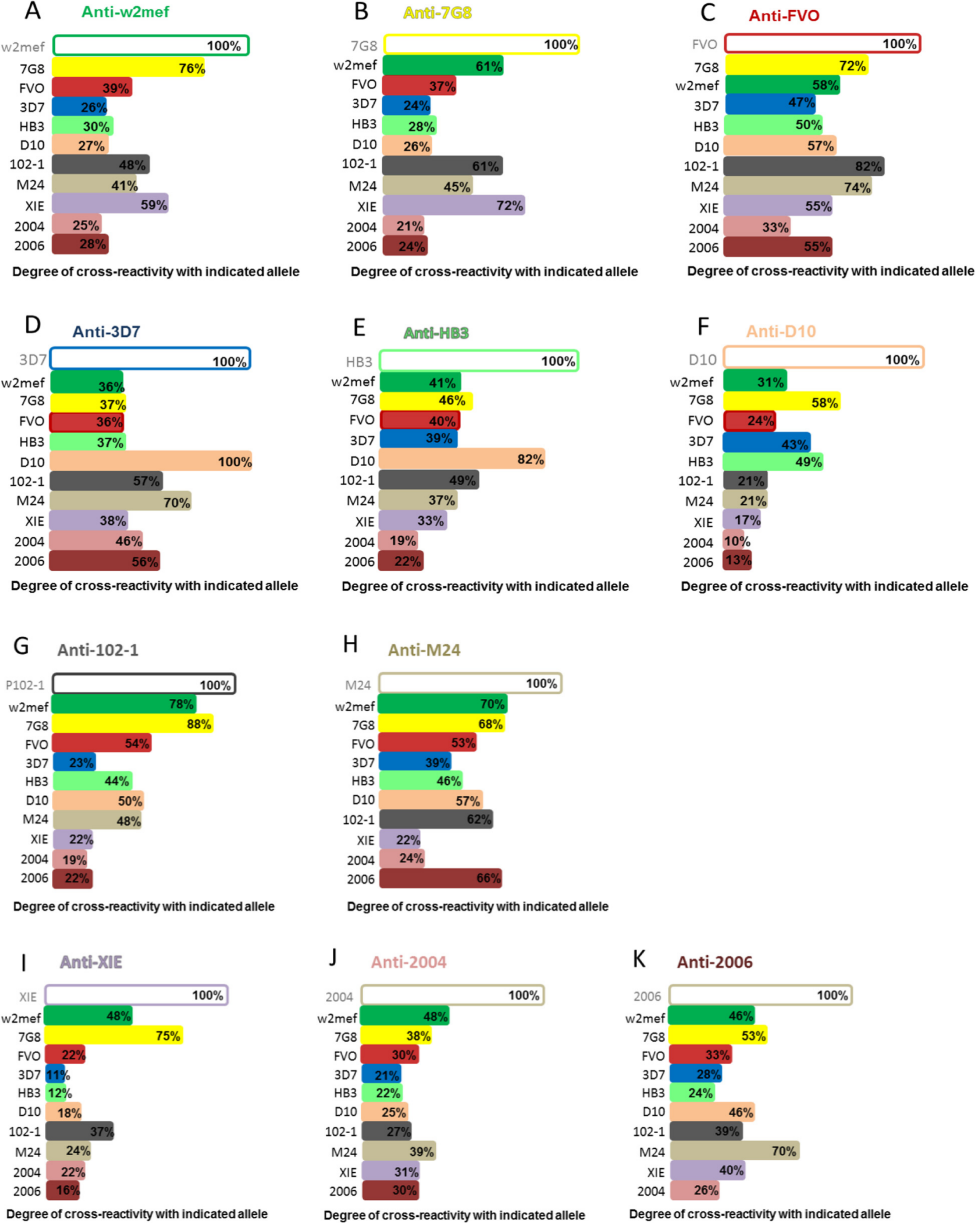
**Cross-reactivity of human AMA1 antibodies measured by competition ELISA.** Human serum pools from a cohort of Papua New Guinean children were tested for naturally acquired antibodies against 11 PfAMA1 variants. Each variant was competed against 10 other AMA1 alleles in competition ELISA and cross-reactivity was determined. Each graph **(A-K)** shows the degree of cross-reactivity observed between antibodies to a particular AMA1 allele and 10 other alleles. For each panel **(A-K)**, the competing antigens used in experiments are those listed on the Y-axis (for example, for panel **A**, W2mef allele was used as the coating antigen, and the Y-axis alleles were used as competitor antigens). AMA1, apical membrane antigen 1.

Initially, we investigated the degree to which AMA1 antibodies were strain-specific or could cross-react with other alleles by testing each allele against the 10 other AMA1 alleles in standard single-antigen competition ELISAs using sera from PNG children. Using competition ELISA, we found that all 11 alleles were cross-reactive at least to some extent with all other alleles (Figure 1). There was considerable variation in the extent of human antibody cross-reactivity for different alleles, and the pattern of cross-reactivity appeared distinct for each allele. AMA1-W2mef antibodies, for instance, were highly cross-reactive with the allele 7G8 (76% cross-reactivity), but showed only limited cross-reactivity with other alleles including 3D7, HB3, D10, 2004 and 2006 (range: 25% to 30%, Figure 1A). Antibodies to AMA1-FVO showed a high degree of cross-reactivity overall, with cross-reactivity higher than 50% to W2mef, HB3, D10, XIE and 2006 alleles, greater than 70% to 7G8 and M24, and 82% to 102-1 (Figure 1C). In contrast cross-reactivity of antibodies to the 102-1 allele was rather low, with 50% or less cross-reactivity to most variants, but high cross-reactivity towards W2mef (78%) and 7G8 (88%, Figure 1G). Antibodies to Pf2004 were highly strain-specific, with less than 50% cross-reactivity to all other alleles (Figure 1J). Interestingly, some alleles showed very high cross-reactivity with specific variants, which was as high as 100% (as seen with 3D7 antibodies towards the D10 allele).

### Greater cross-reactivity of antibodies among adults compared to children

**Figure 2:**
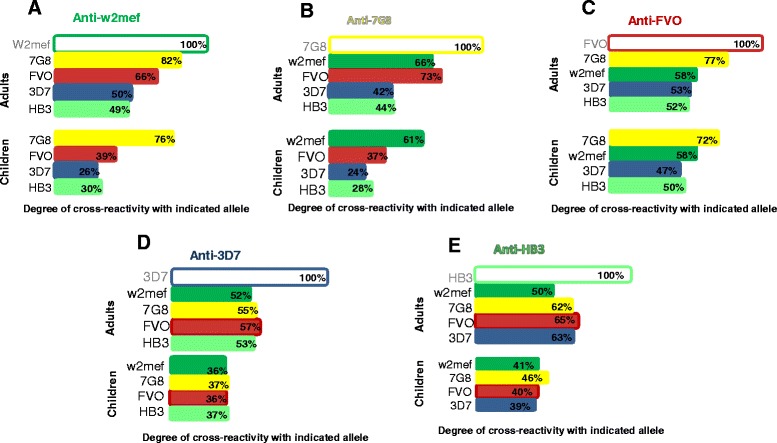
**Cross-reactivity of human AMA1 antibodies in PNG children versus adults.** Each graph **(A-E)** shows cross-reactivity of one specific AMA1 allele. The top part of each graph shows data from a cohort of adults in PNG and the bottom part shows data from a cohort of children in PNG. Each variant **(A-E)** was competed in competition ELISA against four other AMA1 alleles and cross-reactivity was determined. For each graph the color-filled bars in the top and in the bottom part show cross-reactivity between the indicated allele and the variant tested. Cross-reactivity of naturally acquired antibodies in children was slightly lower for all alleles when compared to adults (*P* = 0.002); however, the pattern of cross-reactivity for each allele was similar among children and adults. Data on antibody cross-reactivity for children were extracted from the same dataset as shown in Figure 1. For each figure **(A-E)**, the competing antigens used are those listed on the Y-axis. AMA1, apical membrane antigen 1; PNG, Papua New Guinea.

To assess whether the levels and patterns of antibody cross-reactivity changed over time or with increasing exposure, cross-reactivity of antibodies was compared between pools of serum from adults (n = 42) and children (n = 31) in PNG. For these comparisons we used five representative alleles that had significant differences in sequence: 3D7, HB3, FVO, W2mef, and HB3. Cross-reactivity to all alleles was slightly lower among children compared to adults (*P* <0.01) (Figure 2). However, it was notable that the pattern of cross-reactivity of antibodies to different alleles was very similar among children and adults. The idea that antibody cross-reactivity increases with increasing age and exposure has been previously suggested, but these are the first data to clearly demonstrate this effect.

### Patterns of cross-reactivity are similar over time and between different populations

**Figure 3:**
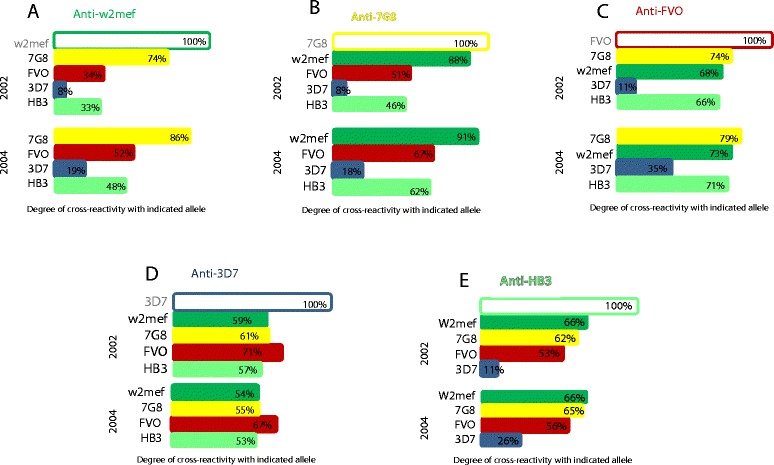
**Patterns of cross-reactivity of AMA1 antibodies in children at two different times.** Each graph **(A-E)** shows antibody cross-reactivity between AMA1 alleles in a cohort of Kenyan children (pooled serum) sampled at two time points, in 2002 and 2004. Each allele was tested in competition ELISA against four other AMA1 alleles and cross-reactivity was determined. The pattern of cross-reactivity and allele-specificity of naturally acquired antibodies of children were very similar at the two time-points; antibodies from older children (2004) tended to have slightly higher cross-reactivity than two years earlier (2002) (*P* <0.05). For each figure **(A-E)**, the competing antigens used in experiments are those listed on the Y-axis. AMA1, apical membrane antigen 1.

We compared the patterns of antibody cross-reactivity to AMA1 among PNG children and Kenyan children. The extended longitudinal nature of the Kenyan cohort allowed us to also assess whether the patterns of antibody cross-reactivity change over time. We prepared pools of samples from children (who were positive for AMA1 antibodies) at two collection time points from the Kenyan cohort, October 2002 and October 2004 (n = 42 samples each point; median age 6.5 for 2002 and 8.3 for 2004; same children’s samples in each pool). In the Kenyan cohort cross-reactivity was compared in serum samples collected in October 2002 and October 2004 (Figure 3). The pattern of cross-reactivity of antibodies to different alleles remained the same over time. AMA1 antibody cross-reactivity tended to be higher at the later time-point (*P* <0.05), but the absolute difference was small (Figure 3A-E). This suggests that significant time and exposure may be required for the development of higher levels of cross-reactivity, such as that seen in adults. Comparing PNG children and adults, or Kenyan children followed longitudinally, the pattern of cross-reactivity remained constant, despite the trend towards an overall increase in cross-reactivity with age. For example, antibodies to W2mef AMA1 showed greatest cross-reactivity to the 7G8 allele, less to FVO and least to 3D7 and HB3, and this pattern was maintained in children and adults (Figure 2A) and when comparing the same children over time (Figure 3A). Patterns of cross-reactivity were similarly maintained for all AMA1 alleles (Figures 1 and 3).

**Figure 4:**
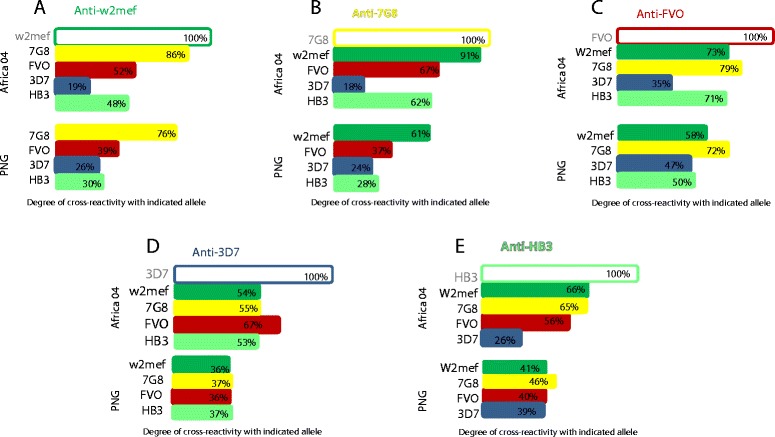
**Patterns of AMA1 antibody cross-reactivity among children from Kenya and PNG.** Each graph **(A-E)** shows antibody cross-reactivity between AMA1 alleles tested by competition ELISA in pooled serum from children from PNG and Kenya (at 2004 time-point). Each variant **(A-E)** was tested against four other AMA1 alleles and cross-reactivity was determined. The pattern of cross-reactivity for each allele was similar in both populations, and cross-reactivity was slightly higher in Kenyan children for all alleles (*P* = 0.017). Data shown here were extracted from the same datasets used in Figures 1 and 3. For each figure **(A-E)**, the competing antigens used in experiments are those listed on the Y-axis. AMA1, apical membrane antigen 1; PNG, Papua New Guinea.

Remarkably, we found that patterns of cross-reactivity among the two geographically distinct populations (PNG and Kenya) were also very similar (Figure 4). Overall, cross-reactivity of antibodies to the five tested alleles was somewhat higher in Kenyan children than in PNG children, but the patterns were comparable. This suggests a similar degree of antigenic diversity of AMA1 in each location, and possibly similar exposure to different alleles, and indicates that sequence or structural differences are the primary determinant of antigenic differences between AMA1 alleles, rather than other population or exposure-specific factors.

### Multiple antigen competition ELISAs

**Figure 5:**
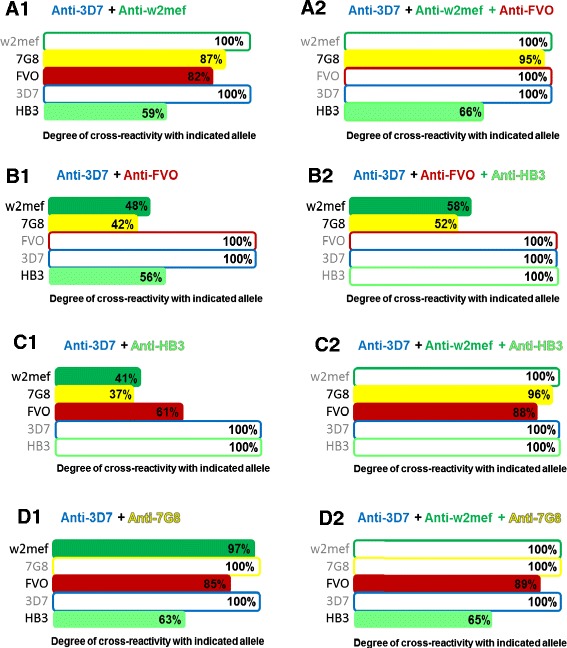
**AMA1 antibody cross-reactivity with multiple competitor alleles in competition ELISAs.** Serum pools prepared from PNG children were tested for cross-reactivity of naturally acquired antibodies among five different PfAMA1 variants in competition ELISA. Competition with either two **(A1 to D1)** or three **(A2 to D2)** other alleles was tested in multiple antigen competition ELISA against the remaining three AMA1 variants to determine cross-reactivity. Enhancement of cross-reactivity by mixtures of two or three competitor alleles was dependent on the specific combination tested. The combination of 3D7 and 7G8 antibodies shows the highest degree of cross-reactivity towards the three other alleles, w2mef, FVO and HB3 **(panel D1)**. Specific combinations of antibodies, that is, 3D7, w2mef and HB3, result in almost complete cross-reactivity towards the remaining AMA1 variants **(panel C2)**. The degree of cross-reactivity observed with a particular combination of competitor alleles indicates the extent to which immunization with that combination might provide coverage against other alleles. A representative selection of double and triple antigen competition ELISAs is shown; all double and triple combination competition ELISAs are shown in Additional file 1: Figure S7. For each panel **(A1-D1, and A2-D2)**, the coating antigens used in experiments are those listed on the Y-axis, and the antigens used for competition are at the top of the figure (for example, for panel **A1**, the coating antigens were W2mef, 7G8, FVO, 3D7, and HB3; the competitor antigens were 3D7 and W2mef). AMA1, apical membrane antigen 1; PNG, Papua New Guinea.

To further define antigenic diversity and relatedness of AMA1 alleles, we developed a novel approach of competition ELISAs with combinations of several competitor AMA1 alleles, which we term MACE. This method allowed us to understand the combined capacity of cross-reactivity of antibodies to two or more AMA1 alleles. Initially we focused on five alleles that differed in sequence (3D7, FVO, HB3, W2mef and 7G8), using combinations of two, three or four alleles as competitors, with PNG children. Combinations were not chosen with regard to their sequence relatedness, instead all possible combinations were tested in order to determine antigenic differences and overlap, and initially identify which combinations could provide the maximum coverage of AMA1 diversity. A high degree of cross-reactivity achieved by a certain combination was taken to indicate that those alleles each had a different spectrum of reactivity and so would produce broader cross-reactivity when combined. Results of these studies could, therefore, improve the understanding of antigenic relatedness of different AMA1 alleles and guide selection of AMA1 alleles to be included in a multi-allele vaccine (Figure 5 shows selected examples using combinations of two and three alleles as competitors).

In some assays, we found that a combination of three heterologous alleles could compete almost as successfully as the homologous allele, indicating that most antibodies to the coating AMA1 allele cross-reacted with one or more of the competitors (for example, 3D7, W2mef and HB3 in Figure 5; Additional file 1: Figures S7 and S8). In contrast, for other combinations the addition of the third heterologous allele showed little additional benefit over two competitors, most likely because antibodies reactive with this allele were already binding the other competitor alleles (for example, adding HB3 to 3D7 + FVO, Figure 5B1 and B2). These data were generated using sera from PNG children, but similar results were observed in PNG adults [see Additional file 1: Figure S6]. The complete data set of all double and triple combinations in PNG children can be found in Additional file 1: Figure S7. Multiple competition ELISAs with combinations of two or three alleles were also performed with sera from the Kenyan cohort, with similar results [see Additional file 1: Figure S8]. Once again, similar patterns of cross-reactivity were observed in the PNG and Kenyan cohorts, with slightly higher cross-reactivity in Kenya. The combination of three alleles that showed the broadest cross-reactivity in PNG (3D7, W2mef and HB3) also showed the greatest coverage in Kenya. Not only have we found the same patterns of cross-reactivity in both geographical regions, but also that the same combinations may provide almost complete coverage in terms of cross-reactivity in both cohorts (Figure 5 and Additional file 1: Figure S8). These findings provided the rationale for further analysis to identify combinations of alleles that might cover antigenic diversity of AMA1 that could guide the selection of alleles for inclusion in multi-allele vaccine development.

### Identifying combinations with broad coverage as possible multi-allele vaccine candidates

**Figure 6:**
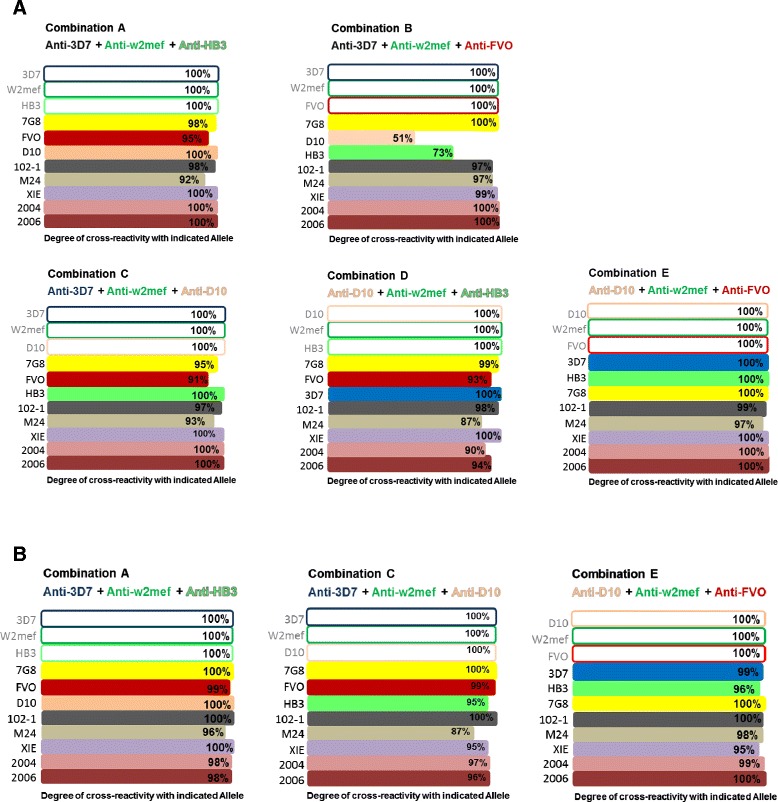
**AMA1 antibody cross-reactivity determined using competition ELISAs with combinations of three AMA1 alleles.** Serum pools from PNG children **(A)** and Kenyan children **(B)** were tested in MACE with selected combinations of three AMA alleles as competitors. Each graph shows a particular combination of three competitor alleles tested against eight other AMA1 alleles. Specific combinations of three AMA1 alleles resulted in high level cross-reactivity against all other alleles, suggesting that immunization with these combinations could provide broad coverage against various AMA1 alleles. Combination E showed close to complete cross-reactivity against all tested AMA1 alleles in both human populations. For each figure the coating antigens used in experiments are those listed on the Y-axis, and the antigens used for competition are noted at the top of the figure. AMA1, apical membrane antigen 1; PNG, Papua New Guinea.

To identify allele combinations that would cover most AMA1 diversity, selected combinations of three antigens were tested against eleven available AMA1 alleles using sera from the PNG cohort. Combinations were selected based on: 1) the extent of cross-reactivity observed between alleles in standard competition ELISAs (Figure 1); and 2) cross-reactivity with combinations of two or three antigens against five alleles in the PNG cohort (Figure 5). We found that Combination A consisting of alleles 3D7, W2mef and HB3 gave cross-reactivity of 92% to 100% across all 11 alleles (Figure 6A). Combination C (3D7, W2mef and D10) showed cross-reactivity of 91% to 100%, Combination D (D10, W2mef and HB3) 87% to 100% and Combination E (D10, W2mef and FVO) showed the highest cross-reactivity across all alleles of 97% to 100%. Interestingly, Combination B consisting of 3D7, W2mef and FVO showed a gap of coverage for D10 and HB3. After the principle was established in the PNG cohort, the most promising combinations were selected and tested in the Kenyan cohort (Figure 6B). Combination A (3D7, W2mef and HB3) and combination E (D10, W2mef and FVO) showed broad, high level cross-reactivity in both populations, similar to observations with PNG samples. These results suggest that it may be possible to reduce a multi-allele-vaccine to either of these combinations of three major AMA 1 serotypes.

### Sequence diversity in AMA1 alleles

**Figure 7:**
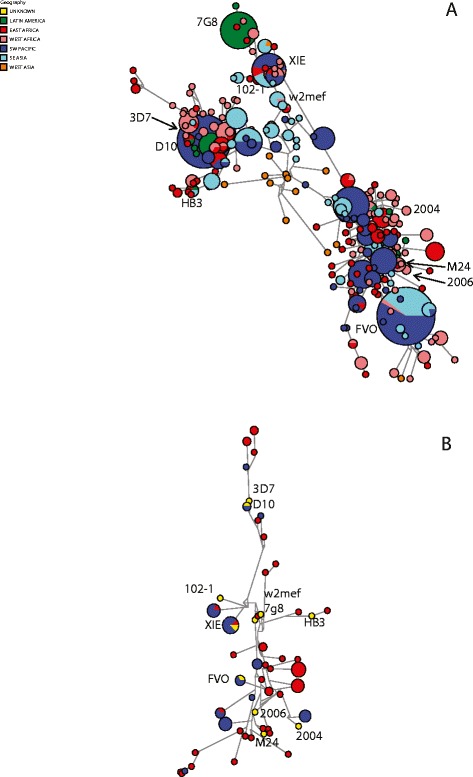
**Evolutionary relationships among AMA1 sequences. (A)** Relationships between 862 global isolates (269 haplotypes) and the 11 reference alleles used in this study (indicated in text). A median joining network with star contraction was drawn in Phylogenetic Network v 4.6.1.2 using default parameters. Node size indicates allele frequency and the geographical origin of sequences is indicated by different colors (key shown). The locations of 11 reference alleles used in this study are indicated throughout the network. **(B)** Evolutionary relationships among AMA1 sequences from the study populations and the 11 reference alleles. Blue, PNG (Madang Province); Red, Kenya (Kilifi District, Yellow (reference alleles). AMA1, apical membrane antigen 1; PNG, Papua New Guinea.

To further support our findings, we performed sequence analyses to evaluate how well the 11 AMA1 reference alleles used in our studies represented global diversity of AMA1, and diversity in our study populations. In the 11 AMA1 alleles used in this study, we found 7 polymorphic amino acid positions in the prodomain and 45 in the ectodomain: 28 in domain I, 8 in domain II, and 9 in domain III (Additional file 1: Figures S1 and S2). These make up 81% of known polymorphic positions in AMA1 (64 identified from 355 AMA1 sequences, [39]). This initial analysis suggests that our selection of AMA1 alleles is broadly representative of global AMA1 diversity. To assess this in more detail, we performed phylogenetic network analyses (median-joining networks) to visualize network trees of evolutionary relationships between different haplotypes. For comparison to our 11 reference alleles, we included 873 AMA1 sequences sourced from all malaria-endemic regions globally, which were found to comprise 269 distinct haplotypes including several high frequency haplotypes (Figure 7A). Our 11 alleles were distributed throughout the network of global sequences, suggesting that they are broadly representative of the global diversity in AMA1 (Figure 7A). The analysis also indicates that there is little evidence of geographic clustering of related sequences, consistent with previous studies, and that global AMA1 sequences group into a small number of major clusters. We performed a similar analysis restricted to including only sequences from our study populations of Madang Province, PNG, and Kilifi District, Kenya (Figure 7B). Our 11 AMA1 reference alleles were again evenly distributed throughout the network, indicating they are representative of the diversity present in our study populations, which is reflected in our serologic data. Sequences from PNG and Kenya were distributed through the networks indicating substantial overlap in the distribution of alleles and polymorphisms in the two populations.

### Relationship between sequence differences in AMA1 alleles and antibody cross-reactivity

**Figure 8:**
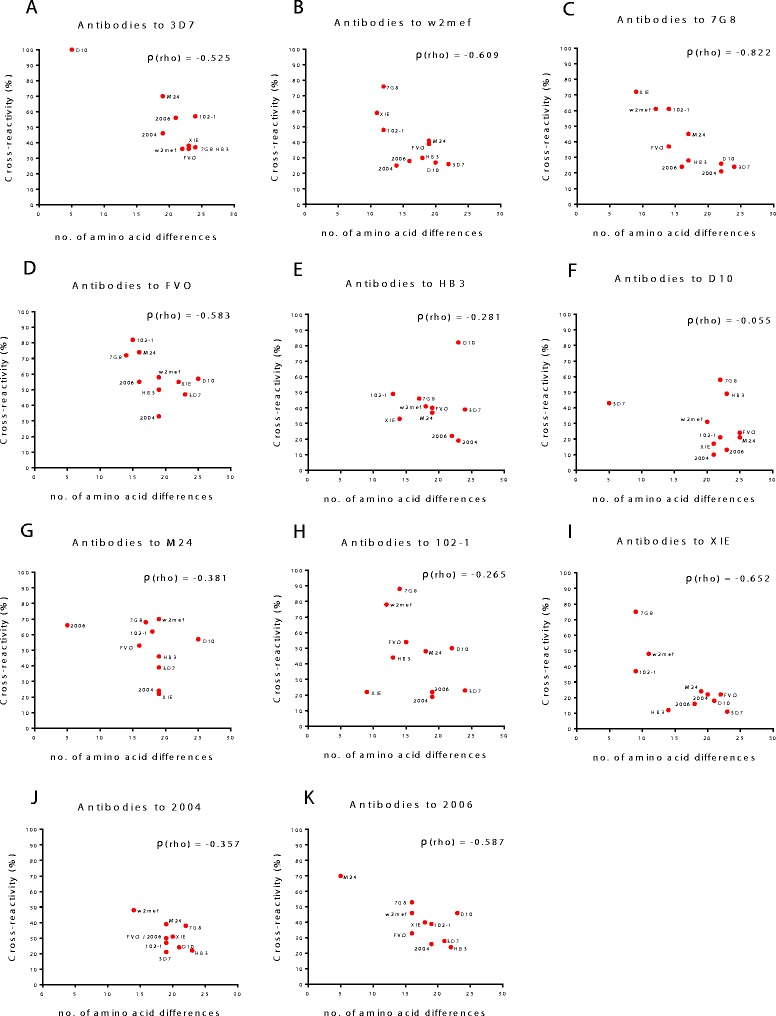
**Relationship between sequence polymorphism and antibody cross-reactivity between AMA1 alleles.** Panels **A-K** show the relationship between the level of antibody cross-reactivity between AMA1 alleles measured by competition ELISA and the number of amino acid differences between the AMA1 alleles across Domains I, II and III. Each panel represents the relationship between a single AMA1 allele to the other 10 AMA1 alleles tested. For example: Panel **A** shows the degree of cross-reactivity that antibodies to 10 other AMA1 alleles have to 3D7 AMA1 by ELISA and the degree of amino acid similarity which each of the 10 alleles have to 3D7 AMA1. Significant negative correlations were seen between cross-reactivity and number of sequence differences for antibodies to 7G8 (Spearman’s ρ (rho) = -0.822, *P* = 0.0047) and XIE (ρ = -0.652, *P* = 0.0438). Negative correlations of borderline statistical significance were observed for antibodies to W2mef (ρ = -0.609, *P* = 0.0667), FVO (ρ = -0.583, P = 0.0806) and 2006 (ρ = -0.587, *P* = 0.0806). No significant correlations were observed for any other alleles. Data on antibody cross-reactivity were obtained from testing PNG children.

To investigate the relationship between antigenic diversity and overall sequence diversity, we related antibody cross-reactivity to the number of sequence differences between each of the AMA1 alleles. For each allele, we correlated the level of antibody cross-reactivity versus the number of polymorphisms in amino acid sequence between alleles across Domain I, II and III of each allele when compared to the other variants (Figure 8 and Additional file 1: Figure S2). Antibody cross-reactivity was generally not strongly related to the number of sequence differences between two alleles, but correlations between sequence differences and antibody cross-reactivity varied for the different alleles. Significant negative correlations were seen between levels of cross-reactivity and number of sequence differences for antibodies to 7G8 (Spearman’s ρ (rho) = -0.822, *P* = 0.0047) and XIE (ρ = -0.652, *P* = 0.0438). Negative correlations of borderline statistical significance were observed for antibodies to W2mef (ρ = -0.609, *P* = 0.0667), FVO (ρ = -0.583, *P* = 0.0806) and 2006 (ρ = -0.587, *P* = 0.0806). No significant correlations were observed for the other alleles. At times, cross-reactivity was high despite a high number of sequence differences between alleles (for example, HB3 and D10, Figure 8E). These findings show that although sequence polymorphisms are related to antibody cross-reactivity and allele-specificity, the overall sequence identity between alleles is not strongly predictive of the extent of antigenic relatedness between alleles.

**Figure 9:**
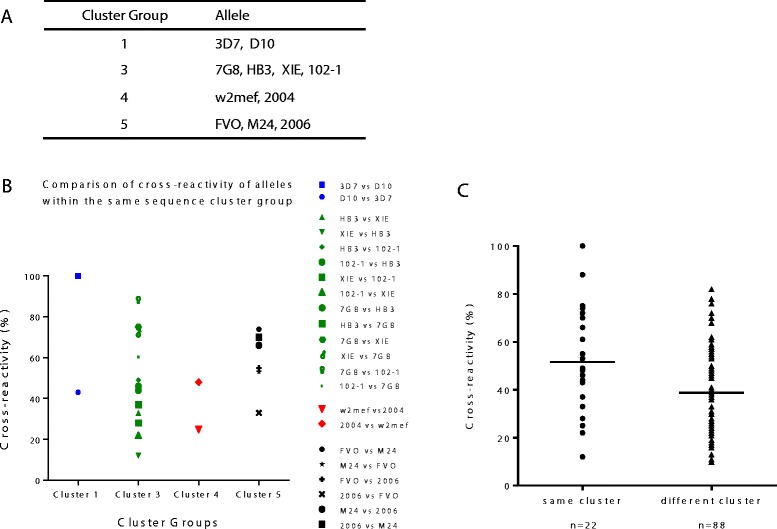
**Analysis of antibody cross-reactivity within amino sequence cluster groups of AMA1 alleles. A)** Classification of the 11 AMA1 alleles into the sequence cluster groups described by Duan *et al*. [35] (based on the sequence including domains I, II and III (excluding the prodomain)). **B)** Antibody cross-reactivity measured by competition ELISA for AMA1 alleles within each cluster group. Alleles within each group were compared pairwise with each other. **C)** Level of antibody cross-reactivity between AMA1 alleles within the same cluster group, compared to cross-reactivity between AMA1 alleles in different clusters - a total of 88 pair-wise comparisons of cross-reactivity was made. The median cross-reactivity of alleles (bar) within the same sequence cluster group was only slightly higher compared to cross-reactivity of alleles from different clusters (p = 0.012; median cross-reactivity was 48.5% for same cluster and 37% for different cluster). Data on antibody cross-reactivity were obtained from testing PNG children. AMA1, apical membrane antigen 1; PNG, Papua New Guinea.

Duan *et al*. grouped 150 AMA1 haplotypes from diverse locations into six groups using a phylogenetic clustering algorithm, and proposed that these groupings based on sequence might provide an indication of immunological cross-reactivity [35,39,40]. To explore this approach as a predictor of antigenic similarity and differences between alleles, we examined our cross-reactivity data in terms of the six groupings they defined. The 11 alleles used in our study fell into four of the six groups generated by the clustering algorithm (Figure 9A). Cross-reactivity between pairs of alleles within each group was assessed (Figure 9B). Cross-reactivity for pairs of alleles within each cluster group was then compared with cross-reactivity for pairs of alleles from different groups (Figure 9C) (for example, XIE and HB3 are both in cluster three; comparing FVO (cluster five) and 3D7 (cluster one) is an example of comparing heterologous clusters). It was notable that there was a large range in the level of cross-reactivity between alleles within the same cluster and between those in different clusters. Cross-reactivity between alleles in the same sequence cluster was slightly greater than that for alleles from different groups. However, there were many alleles in the same cluster that had limited cross-reactivity, and substantial cross-reactivity was seen between alleles that were in different clusters. These analyses indicate that the sequence clustering approach is not a strong predictor of antigenic differences between alleles defined by human antibodies.

## Discussion

Many targets of naturally-acquired immunity to malaria and leading vaccine candidates are polymorphic, which presents challenges to developing these antigens as vaccines that generate broadly protective immunity against different circulating variants or strains in populations. Understanding the antigenic diversity of polymorphic antigens, the level of allele-specific and cross-reactive antibodies, and the relationship between sequence polymorphisms and antigenic escape are essential for advancing vaccine development; however, this knowledge is currently limited for polymorphic malaria antigens. An example of such an antigen is AMA 1, which is a leading malaria vaccine candidate [20,39] and also one of the most polymorphic merozoite antigens. Given the extensive diversity of AMA1 in most populations, single allele immunization is likely to be of limited benefit in endemic areas [20,22]. Here, we investigated antigenic diversity of AMA1 (defined by reactivity to human antibodies) to determine strategies for the development of a potential multi-allele AMA1 vaccine, and more broadly as a model of polymorphic malaria vaccine candidates and the multi-allele vaccine approach. We investigated the antigenic cross-reactivity of a geographically diverse panel of AMA1 alleles with the aim of identifying alleles that might be included in a broadly protective, multi-allele vaccine. Antigenic diversity of AMA1 was found to be surprisingly restricted, despite the large number of haplotypes present in populations. Extensive antibody cross-reactivity against different AMA1 alleles was observed, and results from multiple antigen competition ELISAs indicated that a three-allele vaccine may be sufficient to provide broad coverage against naturally circulating strains, provided the correct alleles are selected. Earlier data from Osier *et al*. indicated that high levels of antibodies to only three AMA1 alleles measured by ELISA were strongly associated with protection from clinical malaria [41]. These results were suggestive that co-acquisition of different allele specific antibodies may produce cross-protection to a larger number of naturally-circulating strains. Until now, there has been little direct evidence to support this interpretation. Our study on cross-reactivity of human anti-AMA1 antibodies helps resolve this question, defining the extent of cross-reactivity towards different alleles.

Competition ELISA experiments demonstrated that antibodies to AMA1 alleles were extensively cross-reactive. Cross-reactivity of more than 70% was observed for some pairs of alleles, suggesting high levels of antigenic relatedness. While other pairs of alleles showed lower cross-reactivity, all combinations displayed at least some cross-reactivity (minimum was 10%). Antibodies that cross-react against different AMA1 alleles probably target shared epitopes (common epitopes that are shared across different alleles) rather than target strictly conserved epitopes (epitopes that are identical on all alleles). Cross-reactive antibodies that protect against different strains of the same pathogen have been well studied in viral diseases, such as influenza or dengue [42-44], and less so in bacteria [45], but there are only limited data on cross-reactive antibodies against individual *P. falciparum* antigens (for example, [31,46]). This is the first study to comprehensively examine the extent to which naturally-acquired human AMA1 antibodies can react with multiple different AMA1 alleles and the most comprehensive analysis of the antigenic diversity, defined by human antibody reactivity, to any merozoite antigen. Although antigenic diversity to AMA1 has also been assessed through the use of antibodies generated in rabbits by immunization, these responses may not be entirely representative of human responses and may differ in nature and specificity. While studies of responses generated by immunization in animal models are informative, reactivity of human antibodies is the most relevant response to evaluate antigenic diversity of AMA1. In addition, the antigenic diversity of AMA1 has been largely evaluated with growth inhibition assays in published studies [24,35,40], and it is not clear whether this is the primary effector mechanism mediating protection. Therefore, we took an approach to measure human antibodies to all epitopes on AMA1.

Competition ELISA experiments comparing cross-reactivity of AMA1 antibodies among malaria-exposed adults and children showed greater antibody cross-reactivity in adults, reflecting a higher level of protective immunity in adults. This observation is supported by an earlier cross-sectional study of AMA1 antibody responses in PNG [13] that showed that of the few individuals identified as having highly allele-specific responses, most were children younger than 10 years of age; however, they did not directly assess antibody cross-reactivity in children versus adults. If conserved or cross-reactive epitopes are less immunogenic than polymorphic epitopes, antibodies might not be produced by younger children either because their immune systems are functionally immature or they have had insufficient exposure to the antigen. Another explanation is suggested by animal immunization studies, which showed that a higher proportion of cross-reactive antibodies to AMA1 was induced by immunization with multiple alleles compared to immunization with a single allele [47,48]. In humans, acquisition of cross-reactive antibodies may also require exposure to a range of alleles, which will be reflected in an increase in the relative proportion of cross-reactive antibodies with cumulative exposure. Future studies to understand variation in the patterns and levels of antibody cross-reactivity to different AMA1 alleles among individuals, and how that relates to age and exposure, will be important and may further inform the selection of alleles for inclusion in a possible multi-allele vaccine. Further studies using an expanded repertoire of AMA1 alleles in single and multiple competition ELISAs, and in additional populations, may also help refine allele-selection in vaccine design.

Importantly, we found very similar patterns of cross-reactivity and allele specificity when comparing AMA1 antibody responses in geographically distinct populations. This suggests that different populations are exposed to a similar repertoire of AMA1 alleles, and that the antigenic properties of AMA1 alleles are the major determinants of cross-reactivity between alleles rather than differences between populations. Furthermore, it is likely that exposure to multiple different alleles in naturally-acquired infections will also influence the acquisition of cross-reactive antibodies. Consistent with our observations, sequence analyses have indicated that while sequence diversity of AMA1 is high, most of the diversity found globally can be identified within a single geographical location [35,49], although there is some geographic clustering of alleles. Analyses suggested that the 11 alleles included in our studies were broadly representative of global diversity. Encouragingly, our findings suggest that the same multi-allele AMA1 vaccine could be effective in different regions and populations.

Multiple competition ELISAs with mixtures of three competitor alleles suggested that antibodies represented in two allele combinations, Combination A (3D7, W2mef and HB3) and Combination E (D10, W2mef and FVO) gave broad, high level cross-reactivity in two geographically distinct human populations (Figure 6), suggesting that a multi-allele-vaccine could probably be reduced to either of these combinations of three major AMA1 serotypes. We propose that the novel MACE approach we have used here could be adopted as an efficient and cost-effective means of testing any polymorphic antigen for its antigenic diversity and the potential of multi-allele approaches in vaccine development. Results from competition ELISA experiments could be used to allocate alleles into potential antigenic or serologic groups that could guide allele selection for AMA1 vaccine development. Alleles with high cross-reactivity against each other by single competition ELISA would be considered to belong to the same serogroup. When two competitor alleles from the same serogroup were combined in double-competition ELISAs, cross-reactivity would not be greatly enhanced compared with results when each allele was used alone as a competitor, reflecting antigenic relatedness between the alleles (see Figure 5). In contrast, combining two competitor alleles from different serogroups would increase cross-reactivity against different alleles. This process guides the selection of alleles to use in combinations.

Several studies have described AMA1 sequence diversity and generated allele-clusters based on sequence similarities using the *Structure* algorithm [22,35,49], with some evidence that these groupings are immunologically relevant. The current study represents the first time AMA1 groupings have been compared to antigenic diversity defined by human antibodies, and we were able to examine how antigenic diversity related to sequence diversity. While there was some relationship between the number of sequence differences between two alleles and the level of antibody cross-reactivity, at times we observed high antibody cross-reactivity between two AMA1 alleles despite a high number of amino acid sequence differences. Our data indicate that broadly assessing sequence differences by the extent of sequence identity between alleles was not a strong or reliable predictor of antibody cross-reactivity between AMA1 alleles, and antigenic diversity is more restricted than sequence diversity. Similarly, grouping sequences using the Bayesian clustering approach used in *Structure* [22,35,49] was also not a strong or reliable predictor of antigenic differences. The lack of a strong relationship between overall sequence differences or haplotype clusters and antigenic differences suggests that only a subset of all polymorphisms are important for defining antigenic differences, that groups of polymorphisms are required for significant antigenic differences, or that some polymorphisms play only a minor role in influencing antigenic differences between alleles. For some other polymorphic malaria antigens, results have suggested that antigenic diversity can be relatively restricted despite significant sequence diversity (for example, EBA175, var2csa [31,50,51]). A previous study identified a polymorphic cluster (C1-L) in AMA1 important for escape from vaccine-induced rabbit antibodies; we found that differences in this region, on their own, were not a good predictor of the antigenic differences we measured here (data not shown). Identifying the most important residues that determine antigenic differences and facilitate immune escape is an important focus for future research; this knowledge would enable the development of sequence-based algorithms that better predict antigenic properties of alleles and would be valuable for application in AMA1 vaccine trials. Recent data from growth inhibition assays using rabbit antisera raised against different AMA1 alleles [24] support these conclusions, demonstrating that the extent of sequence identity between alleles was weakly and inconsistently predictive of antibody cross-inhibitory activity in growth inhibition assays. Furthermore, a mixture of antibodies to four AMA1 alleles was sufficient to inhibit growth of a diverse array of *P. falciparum* isolates [24]. Detailed evaluation of antigenic diversity is crucial for defining which alleles should be included in a multi-allele AMA1 vaccine.

AMA1 antibodies examined here were acquired from natural exposure, and future studies are needed to further evaluate the specificity and cross-reactivity of responses generated by human immunization to support the multi-allele vaccine approach. Immunization of rabbits with a mixture of AMA1 alleles induced a relatively greater proportion of broadly cross-reactive antibodies than single allele immunization [47]; multi-allele immunization appears to shift the antibody responses to epitopes that are conserved or have limited diversity and, therefore, antibodies have greater cross-reactivity than those generated by single-allele immunization [48]. This provides supporting evidence for the multi-allele vaccine approach. However, whether this finding holds true in humans using less potent adjuvants needs to be determined. Supporting these findings, a prior study demonstrated good immunogenicity of a multi-allele AMA1 vaccine in macaques [52]. This vaccine was comprised of AMA1 proteins based on three synthetic sequences (DiCo) that aimed to cover sequence diversity; vaccine-induced antibodies had similar reactivity by ELISA to four different alleles tested (FVO, HB3, 3D7 and CAMP alleles) and gave significant growth inhibition against the three isolates tested (FCR3, HB3 and NF54). Another rabbit immunization study [40] used mixtures of recombinant AMA1 proteins representing each of the cluster groups defined by Duan *et al*. [35] and showed that a five-allele mixture was sufficient to generate antibodies with broad growth inhibitory activity against different isolates. Our findings are in general agreement with these results and, given the differences between humans and experimental animal models in nature and specificity of antibodies, our study provides valuable additional information to guide selection of allele-combinations for human trials and predict vaccine coverage in malaria-endemic settings rather than relying only on small animal immunization studies; findings from different approaches need to be used together to inform vaccine design.

A bi-allele vaccine consisting of recombinant 3D7 and FVO AMA1 alleles was shown to induce antibodies to both alleles in a clinical trial [53,54]. Vaccine-induced antibodies showed cross-reactivity against a non-vaccine AMA1 allele, although the magnitude of response was lower than that against vaccine alleles [53]. Unfortunately, this vaccine showed no efficacy in a phase II trial in Malian children [55]; subsequent investigation found no evidence of strain-specific efficacy (although the sample size was small), and it was suggested that the lack of efficacy was probably because the vaccine formulation was insufficiently immunogenic [56]. Interestingly, the combination of 3D7 and FVO alleles (as used in this vaccine) showed only limited cross-reactivity against different alleles in our study, highlighting the need to carefully select alleles for inclusion in a multi-allele vaccine, with consideration to be given to antigenic relatedness as well as prevalence of circulating alleles in the target population.

Although antibodies measured by ELISA, as performed in our studies, do not directly assess antibody function, they do appear to be a good correlate of functional activity. Antibody levels by ELISA correlate with growth-inhibitory activity in animal and human studies [19,36,40], and antibodies to functional invasion-inhibitory epitopes of AMA1 correlated with total antibody reactivity to AMA1 by ELISA [36]. Equally, levels of antibody reactivity to circumsporozoite proteins measured by ELISA appear to be a good correlate of protection with the RTS,S vaccine [57,58]. Correlations of protection for AMA1 vaccines are not yet available. Further studies that are able to demonstrate comparable levels of cross-reactivity between functional human antibodies to AMA1 alleles (for example, using growth inhibition assays) would provide additional support for the development of a multi-allele AMA1 vaccine, and further research to define the functional activity of AMA1 antibodies is needed. The extent to which human immunization with a multi-allele AMA1 vaccine could protect against naturally circulating *P. falciparum* strains needs to be formally assessed in clinical trials; however, our approach represents a useful tool to predict this coverage.

## Conclusions

In conclusion, AMA1 has been a strong vaccine candidate for many years, but its extensive sequence polymorphism and the limited efficacy of mono- or bi-valent formulations in clinical trials has presented challenges for developing it as an effective vaccine. It has not been clear whether protection would need to be provided by a large repertoire of allele-specific antibodies or antibodies that are able to cross-react with multiple alleles. Here, we have shown extensive cross-reactivity of naturally-acquired antibodies between alleles of AMA1. Whereas sequence polymorphism in AMA1 is high, antigenic diversity is surprisingly restricted, suggesting the feasibility of a multi-allele AMA1 vaccine. Selected combinations of three AMA1 alleles showed high-level, broad cross-reactivity against a range of AMA1 alleles. While further studies with additional alleles are needed to consolidate our findings and further define the alleles for vaccine inclusion, and subsequent testing of allele combinations in clinical trials is needed, our results support the development of AMA1 as a multi-allele vaccine, possibly in combination with other antigens to maximize protective efficacy against malaria. These findings are also broadly relevant to numerous malaria vaccine candidates that are polymorphic, supporting the concept of multi-allele vaccines as a feasible approach and indicating that antigenic diversity may be much lower than predicted by sequence analyses. Our novel MACE method to define antigenic differences and serogroups could be valuable for examining other polymorphic antigens, to characterize antigenic diversity and assess the potential of antigens as future vaccine candidates.

## Additional file

Additional file 1: Figure S1. Amino acid sequence alignment of the 11 AMA1 reference alleles. **Figure S2.** Differences in amino acid sequence between 11 AMA1 alleles. **Figure S3.** Optimising competing AMA1 antigen concentration. **Figure S4.** Example of results from competition ELISA presented as degree of cross-reactivity. **Figure S5.** Antibody cross-reactivity to different AMA1 alleles using individual samples versus a pool of samples. **Figure S6.** AMA1 antibody cross-reactivity with multiple competitor alleles in competition ELISAs with PNG adults. **Figure S7.** AMA1 antibody cross-reactivity with multiple competitor alleles in competition ELISAs with PNG children. **Figure S8.** AMA1 antibody cross-reactivity with multiple competitor alleles in competition ELISAs using samples from the Kenyan cohort.
