# Supplementary material for: Limited antigenic diversity of Plasmodium falciparum apical membrane antigen 1 supports the development of effective multi-allele vaccines
Source: BMC Med. 2014 Oct 16;12:183. doi: 10.1186/s12916-014-0183-5 (PMC4212128; doi:10.1186/s12916-014-0183-5)
Supplement: Additional file 1: Figure S1. — Amino acid sequence alignment of the 11 AMA1 reference alleles. Figure S2. Differences in amino acid sequence between 11 AMA1 alleles. Figure S3. Optimising competing AMA1 antigen concentration. Figure S4. Example of results from competition ELISA presented as degree of cross-reactivity. Figure S5. Antibody cross-reactivity to different AMA1 alleles using individual samples versus a pool of samples. Figure S6. AMA1 antibody cross-reactivity with multiple competitor alleles in competition ELISAs with PNG adults. Figure S7. AMA1 antibody cross-reactivity with multiple competitor alleles in competition ELISAs with PNG children. Figure S8. AMA1 antibody cross-reactivity with multiple competitor alleles in competition ELISAs using samples from the Kenyan cohort. [file 12916_2014_183_MOESM1_ESM.doc]

**Additional File 1:**

**Supplementary Figures S1 to S8**

*Limited antigenic diversity of Plasmodium falciparum apical membrane antigen 1 supports the development of effective multi-allele vaccines*

Ulrich Terheggen^1,2^, Damien R. Drew^1^, Anthony N. Hodder^3^,   Nadia J. Cross^1^, Cleopatra K. Mugyenyi^4^, Alyssa E. Barry^3,5^,Robin F. Anders^6^, Sheetij Dutta^7^, Faith H.A Osier^4^, Salenna R. Elliott^1^, Nicolas Senn^8,9^, Danielle I. Stanisic^3,8^, Kevin Marsh^4^, Peter M. Siba^8^, Ivo Mueller^3,5,8^, Jack S. Richards^1,2,10^,, James G. Beeson^1,2,10^

7G8 MRKLYCVLLLSAFEFTYMINFGRGQNYWEHPYQKSDVYHPINEHREHSKEYEYPLHQEHT 60

PFXIE MRKLYCVLLLSAFEFTYMINFGRGQNYWEHPYQKSDVYHPINEHREHPKEYQYPLHQEHT 60

W2MEF MRKLYCVLLLSAFEFTYMINFGRGQNYWEHPYQKSDVYHPINEHREHPKEYQYPLHQEHT 60

PF102-1 MRKLYCVLLLSAFEFTYMINFGRGQNYWEHPYQKSDVYHPINEHREHPKEYQYPLHQEHT 60

HB3 MRKLYCVLLLSAFEFTYMINFGRGQNYWEHPYQNSDVYRPINEHREHPKEYEYPLHQEHT 60

Pf2006 MRKLYCVLLLSAFEFTYMINFGRGQNYWEHPYQKSDVYHPINEHREHPKEYEYPLHQKHT 60

PFM24 MRKLYCVLLLSAFEFTYMINFGRGQNYWEHPYQNSNVYHPINEHREHPKEYQYPLHQEHT 60

FVO MRKLYCVLLLSAFEFTYMINFGRGQNYWEHPYQKSDVYHPINEHREHPKEYEYPLHQEHT 60

3D7 MRKLYCVLLLSAFEFTYMINFGRGQNYWEHPYQNSDVYRPINEHREHPKEYEYPLHQEHT 60

D10 MRKLYCVLLLSAFEFTYMINFGRGQNYWEHPYQKSDVYHPINEHREHPKEYQYPLHQEHT 60

PF2004 MRKLYCVLLLSAFEFTYMINFGRGQNYWEHPYQKSDVYRPINEHREHPKEYEYPLHQEHT 60

*********************************:*:**:********.***:*****:**

7G8 YQQEDSGEDENTLQHAYPIDHEGAEPAPQEQNLFSSIEIVERSNYMGNPWTEYMAKYDIE 120

PFXIE YQQEDSGEDENTLQHAYPIDHEGAEPAPQEQNLFSSIEIVERSNYMGNPWTEYMAKYDIE 120

W2MEF YQQEDSGEDENTLQHAYPIDHEGAEPAPQEQNLFSSIEIVERSNYMGNPWTEYMAKYDIE 120

PF102-1 YQQEDSGEDENTLQHAYPIDHEGAEPAPQEQNLFSSIEIVERSNYMGNPWTEYMAKYDIE 120

HB3 YQQEDSGEDENTLQHAYPIDHEGAEPAPQEQNLFSSIEIVERSNYMGNPWTEYMAKYDIE 120

Pf2006 YQQEDSGEVENTLQHAYPIDHEGAEPAPQEQNLFSSIEIVERSNYMGNPWTEYMAKYDIE 120

PFM24 YQQEDSGEDENTLQHAYPIDHEGAEPAPQEQNLFSSIEIVERSNYMGNPWTEYMAKYDIE 120

FVO YQQEDSGEDENTLQHAYPIDHEGAEPAPQEQNLFSSIEIVERSNYMGNPWTEYMAKYDIE 120

3D7 YQQEDSGEDENTLQHAYPIDHEGAEPAPQEQNLFSSIEIVERSNYMGNPWTEYMAKYDIE 120

D10 YQQEDSGEDENTLQHAYPIDHEGAEPAPQEQNLFSSIEIVERSNYMGNPWTEYMAKYDIE 120

PF2004 YQQEDSGEDENTLQHAYPIDHEGAEPAPQEQNLFSSIEIVERSNYMGNPWTEYMAKYDIE 120

******** ***************************************************

7G8 EVHGSGIRVDLGEDAEVAGTQYRLPSGKCPVFGKGIIIENSNTTFLKPVATGNQDLKDGG 180

PFXIE EVHGSGIRVDLGEDAEVAGTQYRLPSGKCPVFGKGIIIENSKTTFLTPVATENQDLKDGG 180

W2MEF EVHGSGIRVDLGEDAEVAGTQYRLPSGKCPVFGKGIIIENSNTTFLTPVATGNQYLKDGG 180

PF102-1 EVHGSGIRVDLGEDAEVAGTQYRLPSGKCPVFGKGIIIENSNTTFLTPVATENKDLKDGG 180

HB3 KVHGSGIRVDLGEDAEVAGTQYRLPSGKCPVFGKGIIIENSKTTFLTPVATENQDLKDGG 180

Pf2006 EVHGSGIRVDLGEDAEVAGTQYRLPSGKCPVFGKGIIIENSNTTFLTPVATENQDLKDGG 180

PFM24 EVHGSGIRVDLGEDAEVAGTQYRLPSGKCPVFGKGIIIENSNTTFLTPVATENQDLKDGG 180

FVO EVHGSGIRVDLGEDAEVAGTQYRLPSGKCPVFGKGIIIENSNTTFLKPVATGNQDLKDGG 180

3D7 EVHGSGIRVDLGEDAEVAGTQYRLPSGKCPVFGKGIIIENSNTTFLTPVATGNQYLKDGG 180

D10 EVHGSGIRVDLGEDAEVAGTQYRLPSGKCPVFGKGIIIENSNTTFLTPVATGNQYLKDGG 180

PF2004 EVHGSGIRVDLGEDAEVAGTQYRLPSGKCPVFGKGIIIENSNTTFLTPVATVNQDLKDGG 180

:****************************************:****.**** *: *****

7G8 FAFPPTNPLISPMTLDHMRDFYKNNEYVKNLDELTLCSRHAGNMNPDNDKNSNYKYPAVY 240

PFXIE FAFPPTEPLMSPMTLDDMRRFYKDNEYVKNLDELTLCSRHAGNMNPDNDKNSNYKYPAVY 240

W2MEF FAFPPTKPLMSPMTLDDMRLLYKDNEDVKNLDELTLCSRHAGNMNPDNDKNSNYKYPAVY 240

PF102-1 FAFPPTEPLMSPMTLDDMRRFYKDNEYVKNLDELTLCSRHAGNMNPDNDKNSNYKYPAVY 240

HB3 FAFPPTEPLISPMTLDQMRHLYKDNEYVKNLDELTLCSRHAGNMNPDNDKNSNYKYPAVY 240

Pf2006 FAFPPTKPLMSPMTLDQMRDFYKNNEYVKNLDELTLCSRHAGNMNPDNDENSNYKYPAVY 240

PFM24 FAFPPTKPLMSPMTLDQMRDFYKNNEYVKNLDELTLCSRHAGNMNPDNDENSNYKYPAVY 240

FVO FAFPPTNPLISPMTLNGMRDFYKNNEYVKNLDELTLCSRHAGNMNPDNDKNSNYKYPAVY 240

3D7 FAFPPTEPLMSPMTLDEMRHFYKDNKYVKNLDELTLCSRHAGNMIPDNDKNSNYKYPAVY 240

D10 FAFPPTEPLMSPMTLDEMRHFYKDNKYVKNLDELTLCSRHAGNMIPDNDKNSNYKYPAVY 240

PF2004 FAFPPTKPLMSPMTLDDMRLLYKDNEDVKNLDELTLCSRHAGNMIPDNDKNSNYKYPAVY 240

******:**:*****: ** :**:*: ***************** ****:**********

7G8 DYNDKKCHILYIAAQENNGPRYCNKDESKRNSMFCFRPAKDKSFQNYTYLSKNVVDNWEK 300

PFXIE DYNDNKCHILYIAAQENNGPRYCNKDESKRNSMFCFRPAKDKSFQNYTYLSKNVVDNWEK 300

W2MEF DYNDKKCHILYIAAQENNGPRYCNKDESKRNSMFCFRPAKDKSFQNYTYLSKNVVDNWEE 300

PF102-1 DYNDKKCHILYIAAQENNGPRYCNKDQSKRNSMFCFRPAKDKLFENYTYLSKNVVDNWEE 300

HB3 DYEDKKCHILYIAAQENNGPRYCNKDESKRNSMFCFRPAKDKLFENYTYLSKNVVDNWEE 300

Pf2006 DYKDKKCHILYIAAQENNGPRYCNKDQSKRNSMFCFRPAKDKSFQDYTYLSKNVVDNWEK 300

PFM24 DYKDKKCHILYIAAQENNGPRYCNKDQSKRNSMFCFRPAKDKLFENYTYLSKNVVHNWEK 300

FVO DYNDKKCHILYIAAQENNGPRYCNKDQSKRNSMFCFRPAKDKLFENYTYLSKNVVDNWEE 300

3D7 DDKDKKCHILYIAAQENNGPRYCNKDESKRNSMFCFRPAKDISFQNYTYLSKNVVDNWEK 300

D10 DDKDKKCHILYIAAQENNGPRYCNKDESKRNSMFCFRPAKDISFQNYTYLSKNVVDNWEK 300

PF2004 DDKDKKCHILYIAAQENNGPRYCNKDESKRNSMFCFRPAKDKLFENYTYLSKNVVDNWEK 300

* :*:*********************:************** *::*********.***:

7G8 VCPRKNLENAKFGLWVDGNCEDIPHVNEFSANDLFECNKLVFELSASDQPKQYEQHLTDY 360

PFXIE VCPRKNLENAKFGLWVDGNCEDIPHVNEFSANDLFECNKLVFELSASDQPKQYEQHLTDY 360

W2MEF VCPRKNLENAKFGLWVDGNCEDIPHVNEFSANDLFECNKLVFELSASDQPKQYEQHLTDY 360

PF102-1 VCPRKNLENAKFGLWVDGNCEDIPHVNEFSANDLFECNKLVFELSASDQPKQYEQHLTDY 360

HB3 VCPRKNLENAKFGLWVDGNCEDIPHVNEFSANDLFECNKLVFELSASDQPKQYEQHLTDY 360

Pf2006 VCPRKNLENAKFGLWVDGNCEDIPHVNEFSANDLFECNKLVFELSASDQPKQYEQHLTDY 360

PFM24 VCPRKNLQNAKFGLWVDGNCEDIPHVNEFSANDLFECNKLVFELSASDQPKQYEQHLTDY 360

FVO VCPRKNLENAKFGLWVDGNCEDIPHVNEFSANDLFECNKLVFELSASDQPKQYEQHLTDY 360

3D7 VCPRKNLQNAKFGLWVDGNCEDIPHVNEFPAIDLFECNKLVFELSASDQPKQYEQHLTDY 360

D10 VCPRKNLQNAKFGLWVDGNCEDIPHVNEFSAIDLFECNKLVFELSASDQPKQYEQHLTDY 360

PF2004 VCPRKNLENAKFGLWVDGNCEDIPHVNKFSANDLFECNKLVFELSASDQPKQYEQHLTDY 360

*******:*******************:*.* ****************************

7G8 EKIKEGFKNKNASMIKSAFLPTGAFKADRYKSRGKGYNWGNYNRKTQKCEIFNVKPTCLI 420

PFXIE EKIKEGFKNKNASMIKSAFLPTGAFKADRYKSRGKGYNWGNYNRKTQKCEIFNVKPTCLI 420

W2MEF EKIKEGFKNKNASMIKSAFLPTGAFKADRYKSHGKGYNWGNYNRKTQKCEIFNVKPTCLI 420

PF102-1 EKIKEGFKNKNASMIKSAFLPTGAFKADRYKSHGKGYNWGNYNRETQKCEIFNVKPTCLI 420

HB3 EKIKEGFKNKNASMIKSAFLPTGAFKADRYKSRGKGYNWGNYNTETQKCEIFNVKPTCLI 420

Pf2006 EKIKEGFKNKNASMIKSAFLPTGAFKADRYKSHGKGYNWGNYNTKTQKCEIFNVKPTCLI 420

PFM24 EKIKEGFKNKNASMIKSAFLPTGAFKADRYKSRGKGYNWGNYNTKTQKCEIFNVKPTCLI 420

FVO EKIKEGFKNKNASMIKSAFLPTGAFKADRYKSHGKGYNWGNYNRETQKCEIFNVKPTCLI 420

3D7 EKIKEGFKNKNASMIKSAFLPTGAFKADRYKSHGKGYNWGNYNTETQKCEIFNVKPTCLI 420

D10 EKIKEGFKNKNASMIKSAFLPTGAFKADRYKSHGKGYNWGNYNTETQKCEIFNVKPTCLI 420

PF2004 EKIKEGFKNKNASMIKSAFLPTGAFKADRYKSHGRGYNWGNYNRKTQKCEIFNVKPTCLI 420

********************************:*:******** :***************

7G8 NNSSYIATTALSHPNEVEHNFPCSLYKDEIKKEIERESKRIKLNDNDDEGNKKIIAPRIF 480

PFXIE NNSSYIATTALSHPNEVEHNFPCSLYKDEIKKEIERESKRIKLNDNDDEGNKKIIAPRIF 480

W2MEF NNSSYIATTALSHPIEVEHNFPCSLYKDEIKKEIERESKRIKLNDNDDEGNKKIIAPRIF 480

PF102-1 NNSSYIATTALSHPNEVEHNFPCSLYKDEIKKEIERESKRIKLNDNDDEGNKKIIAPRIF 480

HB3 NNSSYIATTALSHPNEVENNFPCSLYKDEIKKEIERESKRIKLNDNDDEGNKKIIAPRIF 480

Pf2006 NNSSYIATTALSHPIEVEHNFPCSLYKDEIKKEIERESKRIKLNDNDDEGNKKIIAPRIF 480

PFM24 NNSSYIATTALSHPIEVEHNFPCSLYKDEIKKEIERESKRIKLNDNDDEGNKKIIAPRIF 480

FVO NNSSYIATTALSHPIEVEHNFPCSLYKDEIKKEIERESKRIKLNDNDDEGNKKIIAPRIF 480

3D7 NNSSYIATTALSHPIEVENNFPCSLYKDEIMKEIERESKRIKLNDNDDEGNKKIIAPRIF 480

D10 NNSSYIATTALSHPIEVEHNFPCSLYKNEIMKEIERESKRIKLNDNDDEGNKKIIAPRIF 480

PF2004 NNSSYIATTALSHPIEVEHNFPCSLYKDEIKKEIERESKRIKLNDNDDEGNKKIIAPRIF 480

************** ***:********:** *****************************

7G8 ISDDIDSLKCPCDPEIVSNSTCNFFVCKCVEKRAEVTSNNEVVVKEEYKDEYADIPEHKP 540

PFXIE ISDDIDSLKCPCDPEIVSNSTCNFFVCKCVEKRAEVTSNNEVVVKEEYKDEYADIPEHKP 540

W2MEF ISDDIDSLKCPCDPEIVSNSTCNFFVCKCVEKRAEVTSNNEVVVKEEYKDEYADIPEHKP 540

PF102-1 ISDDIDSLKCPCDPEIVSNSTCNFFVCKCVEKRAEVTSNNEVVVKEEYKDEYADIPEHKP 540

HB3 ISDDKDSLKCPCDPEIVSNSTCNFFVCKCVEKRAEVTSNNEVVVKEEYKDEYADIPEHKP 540

Pf2006 ISDDIDSLKCPCDPEMVSNSTCRFYVCKCVERRAEVTSNNEVVVKEEYKDEYADIPEHKP 540

PFM24 ISDDIDSLKCPCDPEMVSNSTCRFYVCKCVERRAEVTSNNEVVVKEEYKDEYADIPEHKP 540

FVO ISDDKDSLKCPCDPEMVSNSTCRFFVCKCVERRAEVTSNNEVVVKEEYKDEYADIPEHKP 540

3D7 ISDDKDSLKCPCDPEMVSNSTCRFFVCKCVERRAEVTSNNEVVVKEEYKDEYADIPEHKP 540

D10 ISDDKDSLKCPCDPEIVSNSTCNFFVCKCVERRAEVTSNNEVVVKEEYKDEYADIPEHKP 540

PF2004 ISDDKDSLKCPCDPEMVSNSTCRFFVCKCVERRAEVTSNNEVVVKEEYKDEYADIPEHKP 540

**** **********:******.*:******:****************************

7G8 TYDKMKIIIASSAAVAVLATILMVYLYKRKGNAEKYDKMDEPQDYGKSNSRNDEMLDPEA 600

PFXIE TYDKMKIIIASSAAVAVLATILMVYLYKRKGNAEKYDKMDEPQHYGKSNSRNDEMLDPEA 600

W2MEF TYDKMKIIIASSAAVAVLATILMVYLYKRKGNAEKYDKMDEPQHYGKSNSRNDEMLDPEA 600

PF102-1 TYDKMKIIIASSAAVAVLATILMVYLYKRKGNAEKYDKMDEPQHYGKSNSRNDEMLDPEA 600

HB3 TYDNMKIIIASSAAVAVLATILMVYLYKRKGNAEKYDKMDQPQHYGKSNSRNDEMLDPEA 600

Pf2006 TYDNMKIIIASSAAVAVLATILMVYLYKRKGNAEKYDKMDEPQHYGKSNSRNDEMLDPEA 600

PFM24 TYDNMKIIIASSAAVAVLATILMVY-YKRKGNAEKYDKMDQPQDYGKSTSRNDEMLDPEA 599

FVO TYDNMKIIIASSAAVAVLATILMVYLYKRKGNAEKYDKMDQPQHYGKSTSRNDEMLDPEA 600

3D7 TYDKMKIIIASSAAVAVLATILMVYLYKRKGNAEKYDKMDEPQDYGKSNSRNDEMLDPEA 600

D10 TYDKMKIIIASSAAVAVLATILMVYLYKRKGNAEKYDKMDEPQHYGKSNSRNDEMLDPEA 600

PF2004 TYDKMKIIIASSAAVAVLATILMVYLYKRKGNAEKYDKMDEPQHYGKSNSRNDEMLDPEA 600

***:********************* **************:**.****.***********

7G8 SFWGEEKRASHTTPVLMEKPYY 622

PFXIE SFWGEEKRASHTTPVLMEKPYY 622

W2MEF SFWGEEKRASHTTPVLMEKPYY 622

PF102-1 SFWGEEKRASHTTPVLMEKPYY 622

HB3 SFWGEEKRASHTTPVLMEKPYY 622

Pf2006 SFWGEEKRASHTTPVLMEKPYY 622

PFM24 SFWGEEKRASHTTPVLMEKPYY 621

FVO SFWGEEKRASHTTPVLMEKPYY 622

3D7 SFWGEEKRASHTTPVLMEKPYY 622

D10 SFWGEEKRASHTTPVLMEKPYY 622

PF2004 SFWGEEKRASHTTPVLMEKPYY 622

**********************

**Figure S1. Amino acid sequence alignment of the 11 AMA1 reference alleles.** Amino acid sequences from the 11 AMA1 reference alleles included in this study were obtained from the Protein Data Bank (PDB) and sequence alignment performed using online software CLUSTAL 2.1. Sequences include 622 amino acids (aa): signal peptide (positions 1-25) prodomain and Domains I, II and III (position 26-546; total of 522 aa), and transmembrane domain (position 547-622). The star symbol beneath the alignment indicates amino acid identity, dots or blanks indicate di- or polymorphic aa. Amongst these 11 AMA1 alleles, we found in total 52 di- or polymorphic aa positions, 7 in the prodomain and 45 in the ectodomain: 28 in DI, 8 in DII, and 9 in DIII.

| **Alleles** | 3D7 | D10 | 2004 | 7G8 | w2mef | XIE | FVO | M24 | 101-2 | 2006 | HB3 |
| --- | --- | --- | --- | --- | --- | --- | --- | --- | --- | --- | --- |
| 3D7 | — | **1.5%**  **8**  (3/0/1/4) | **3.8%**  **20**  (1/11/6/2) | **5.2%**  **27**  (3/12/6/6) | **4.8%**  **25**  (3/12/4/6) | **5%**  **26** (3/11/6/6) | **4.8%**  **25**  (2/17/3/3) | **5%**  **26**  (3/14/4/5) | **5.2%**  **27**  (3/14/4/6) | **4.8%**  **25**  (4/13/3/5) | **4.6%**  **24**  (0/15/4/5) |
| D10 |  | — | **4.4%**  **23**  (2/12/5/4) | **4.6%**  **24**  (2/13/5/4) | **3.8%**  **20**  (0/13/3/4) | **4%**  **21**  (0/12/5/4) | **5%**  **26**  (1/18/2/5) | **5.2%**  **27**  (2/15/3/7) | **4.2%**  **22**  (0/15/3/4) | **5%**  **26**  (3/14/2/7) | **5%**  **26**  (3/15/3/5) |
| 2004 |  |  | — | **4.6%**  **24**  (2/14/4/4) | **3%**  **16**  (2/8/2/4) | **4.2%**  **22**  (2/12/4/4) | **3.8%**  **20**  (1/15/3/1) | **4.4%**  **23**  (4/12/4/3) | **4%**  **21**  (2/11/4/4) | **4.2%**  **22**  (3/13/3/3) | **4.6%**  **24**  (1/12/6/5) |
| 7G8 |  |  |  | — | **2.7%**  **14**  (2/10/2/0) | **2.1%**  **11**  (2/9/0/0) | **2.9%**  **15**  (1/6/3/5) | **4%**  **21**  (4/10/2/5) | **3%**  **16**  (2/12/2/0) | **3.6%**  **19**  (3/8/3/5) | **3.8%**  **20**  (3/12/2/3) |
| w2mef |  |  |  |  | — | **2.1%**  **11**  (0/9/2/0) | **3.8%**  **20**  (1/13/1/5) | **4%**  **21**  (2/12/2/5) | **2.3%**  **12**  (0/10/2/0) | **3.6%**  **19**  (3/10/1/5) | **4%**  **21**  (3/11/4/3) |
| XIE |  |  |  |  |  | — | **4.4%**  **23**  (1/14/3/5) | **4%**  **21**  (2/12/2/5) | **1.7%**  **9**  (0/7/2/0) | **4%**  **21**  (3/10/3/5) | **3.3%**  **17**  (3/9/2/3) |
| FVO |  |  |  |  |  |  | — | **3.6%**  **19**  (3/11/3/2) | **3%**  **16**  (1/9/1/5) | **3.4%**  **18**  (2/12/2/2) | **4%**  **21**  (2/12/3/4) |
| M24 |  |  |  |  |  |  |  | — | **3.8%**  **20**  (2/9/4/5) | **1.9%**  **10**  (5/4/1/0) | **4.2%**  **22**  (3/11/2/6) |
| 102-1 |  |  |  |  |  |  |  |  | — | **4.2%**  **22**  (3/11/3/5) | **3%**  **16**  (3/8/2/3) |
| 2006 |  |  |  |  |  |  |  |  |  | — | **5%**  **26**  (4/13/3/6) |
| HB3 |  |  |  |  |  |  |  |  |  |  | — |

**Figure S2. Differences in amino acid sequence between 11 AMA1 alleles**

Percentage and total number of amino acid differences between AMA1 alleles are indicated, with number of differences for Pro-Domain, Domains I, II and III shown in brackets. AMA1 has 622 amino acids (aa), 522aa in the prodomain, DI, II, III and 100aa in the signal peptide and transmembrane domain. A total of 64 polymorphic aa positions have been identified in 355 different AMA1 sequences (Remarque et al., 2008): 9 in prodomain, 32 in DI, 11 in DII, 9 in DIII. In the 11 sequences of AMA1 alleles above, we found in total 52 polymorphic aa positions (7 in prodomain, 28 in DI, 8 in DII, and 9 in DIII), showing that the 11 alleles used in this study include 81% of known polymorphisms.

**Figure S3. Optimising competing AMA1 antigen concentration in competition ELISA.** Plates were coated with 3D7 AMA-1 and plasma samples were pre- incubated with homologous antigen, before testing for antibody binding to immobilized AMA1 by ELISA. For all plasma samples tested, 5ug/ml of homologous competitor appeared to achieve maximal inhibition of antibody reactivity to bound AMA-1. Each line represents a different Papua New Guinean plasma sample.

**A**

**B**

Antibody reactivity to AMA1-7G8

**
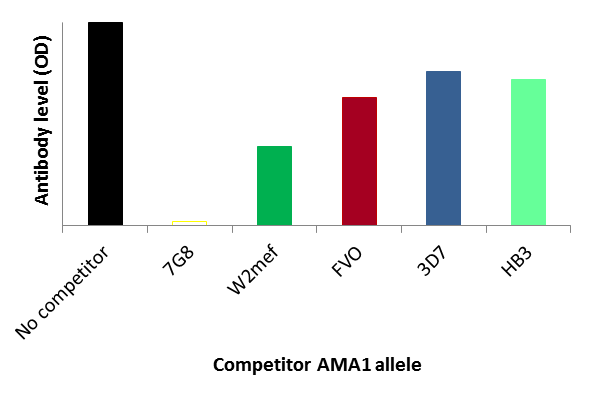
**

**Anti-7G8 Antibodies**

7G8

W2mef

FVO

3D7

HB3

**100%**

**61%**

**37%**

**24%**

**28%**

Degree of cross-reactivity with indicated allele

**Figure S4. Example of results from competition ELISA presented as degree of cross-reactivity.** Cross-reactivity of serum antibodies (using a pool of PNG children’s samples) to the coating AMA1 allele (7G8) is derived from the degree of inhibition (%) observed with each competitor AMA1 allele **(shown in A)** when compared with antibody level measured in the absence of competitor. Results for competition ELISAs performed with AMA1 7G8 allele is shown as an example, with the degree of antibody cross-reactivity shown in (**B**).


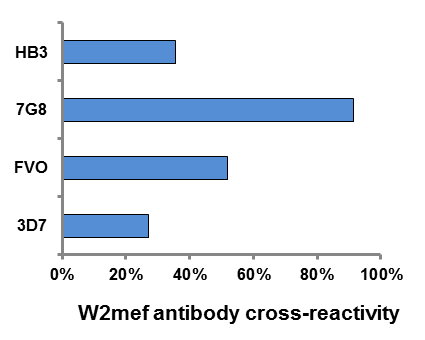

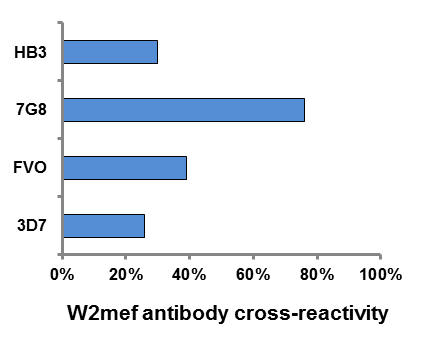


***A***

***B***

**Figure S5. Antibody cross-reactivity to different AMA1 alleles using individual samples versus a pool of samples.** Human serum samples from Papua New Guinean children were tested for the level of antibody cross-reactivity to different AMA1 alleles using competition ELISA. AMA1-W2mef was used as the coating antigen and the competing antigens used in experiments are those listed on the Y-axis. The patterns and extent of cross reactivity of AMA1-W2mef antibodies to four different AMA1 alleles were very similar when individual samples (n=31) were tested (**A**), or when a pool of the same samples was used in the assays (**B**). In (**A**), results shown are the median cross-reactivity of samples tested.

**Anti-3D7 + Anti-w2mef**

**Anti-3D7 + Anti-w2mef + Anti-HB3**

**A1**

**A2**

w2mef

7G8

FVO

3D7

HB3

**100%**

**Degree of crossreaction with indicated Allele**

w2mef

7G8

FVO

3D7

HB3

**100%**

**81%**

**89%**

**76%**

**86%**

**100%**

**100%**

**65%**

**100%**

**Degree of crossreaction with indicated Allele**

**Anti-3D7 + Anti-FVO + Anti-w2mef**

**Anti-3D7 + Anti-FVO**

**B1**

**B2**

w2mef

7G8

FVO

3D7

HB3

**Degree of crossreaction with indicated Allele**

w2mef

7G8

FVO

3D7

HB3

**100%**

**80%**

**85%**

**106%**

**100%**

**100%**

**100%**

**100%**

**84%**

**80%**

**Degree of crossreaction with indicated Allele**

**C1**

**Anti-3D7 + Anti-FVO + Anti-w2mef + Anti- 7G8**

w2mef

7G8

FVO

3D7

HB3

**100%**

**100%**

**100%**

**100%**

**84%**

**Degree of crossreaction with indicated Allele**

**Figure S6. AMA1 antibody cross-reactivity with multiple competitor alleles in competition ELISAs using samples from PNG adults.**  Serum pools prepared from PNG adults were tested for AMA1 antibody cross-reactivity in competition ELISAs using either two (A1-B1) or three (A2-B2) competitor AMA1 alleles. Enhancement of cross-reactivity by mixtures of two or three competitor alleles was dependent on the specific combination tested. Competition with combinations of 4 different alleles did not further enhance cross-reactivity when compared to combinations of three alleles (C1).

**A1**

**A2**

**
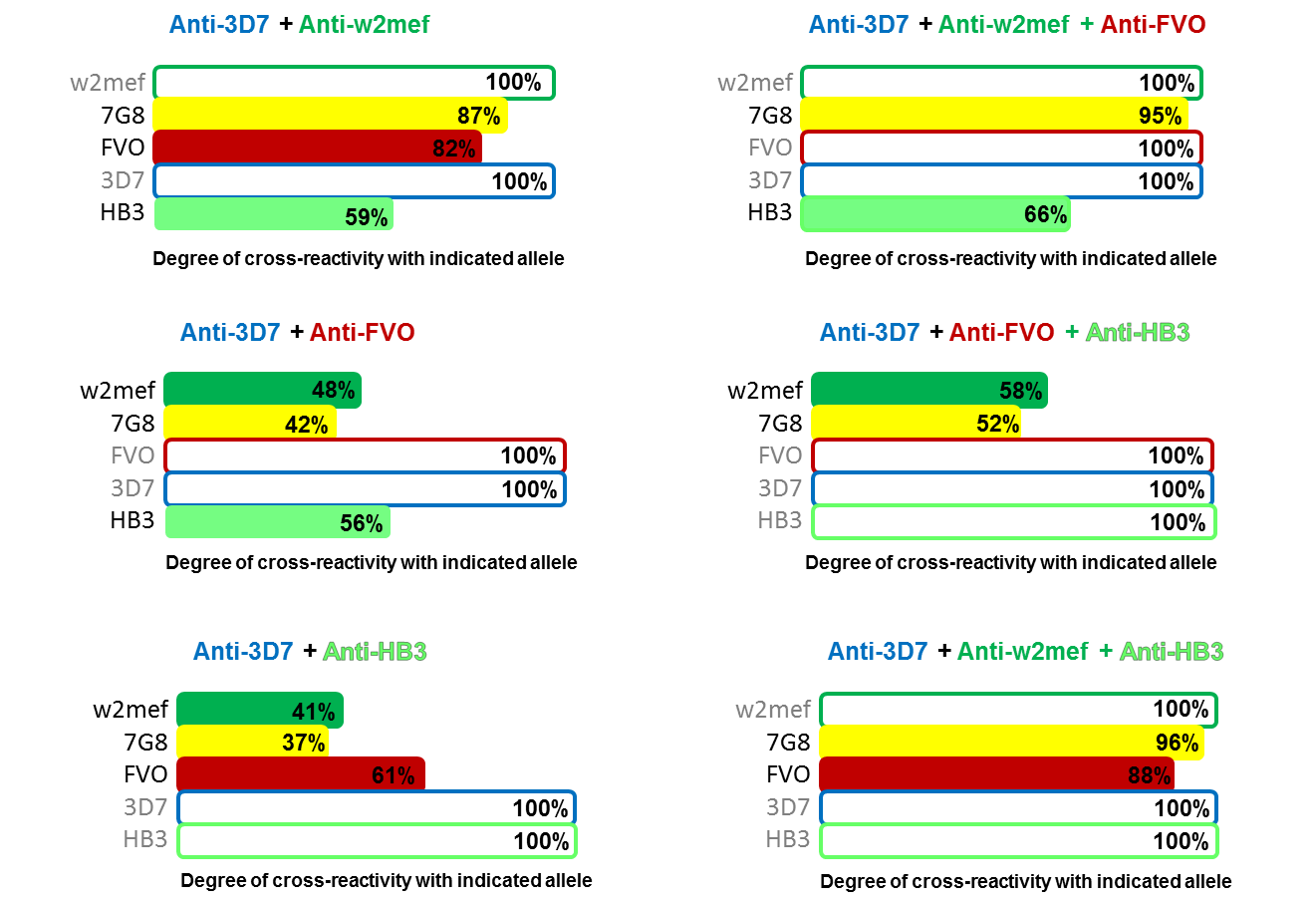
**

**B1**

**C2**

**B2**

**C1**

**D2**

**D1**


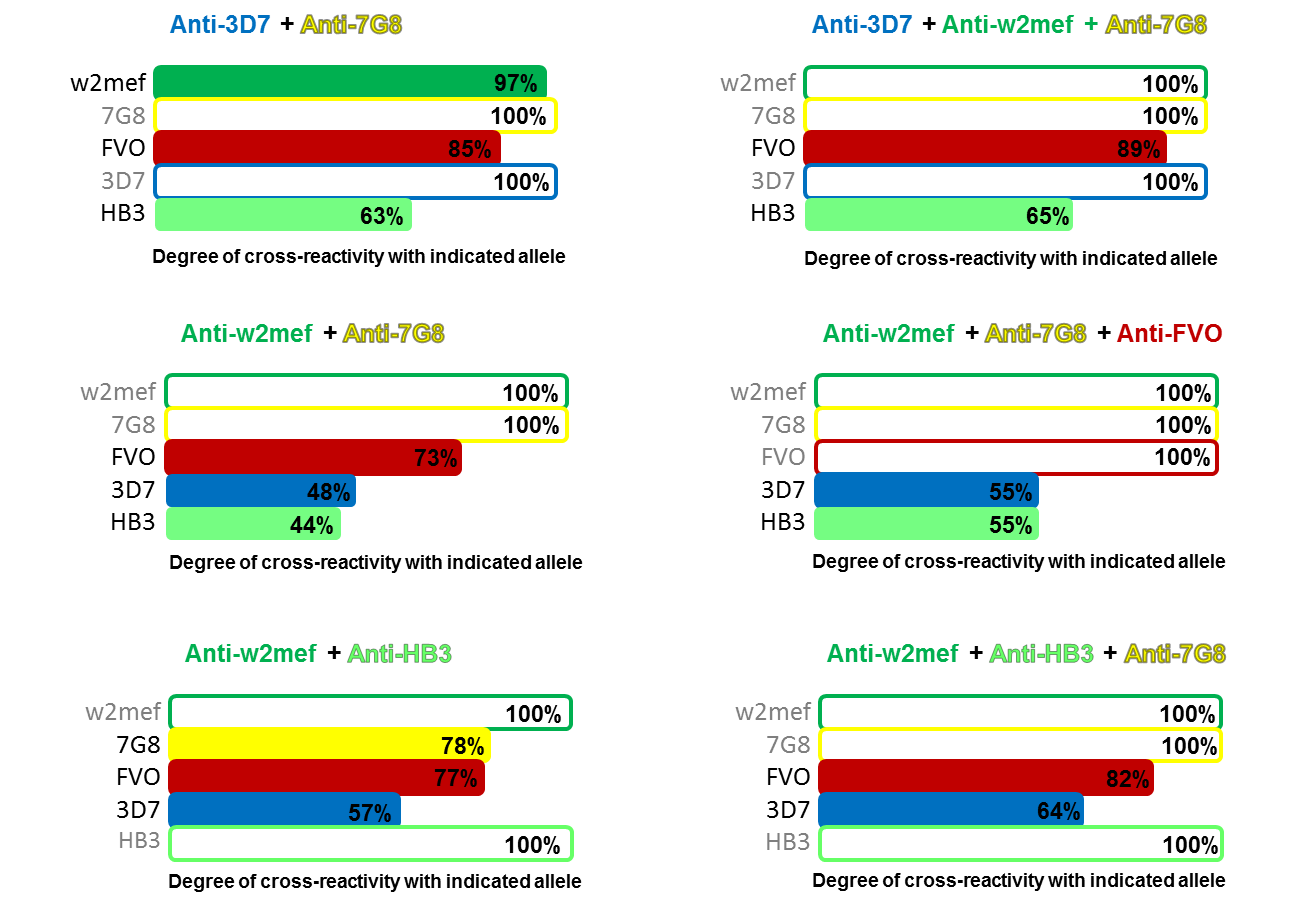


**F2**

**E2**

**F1**

**E1**


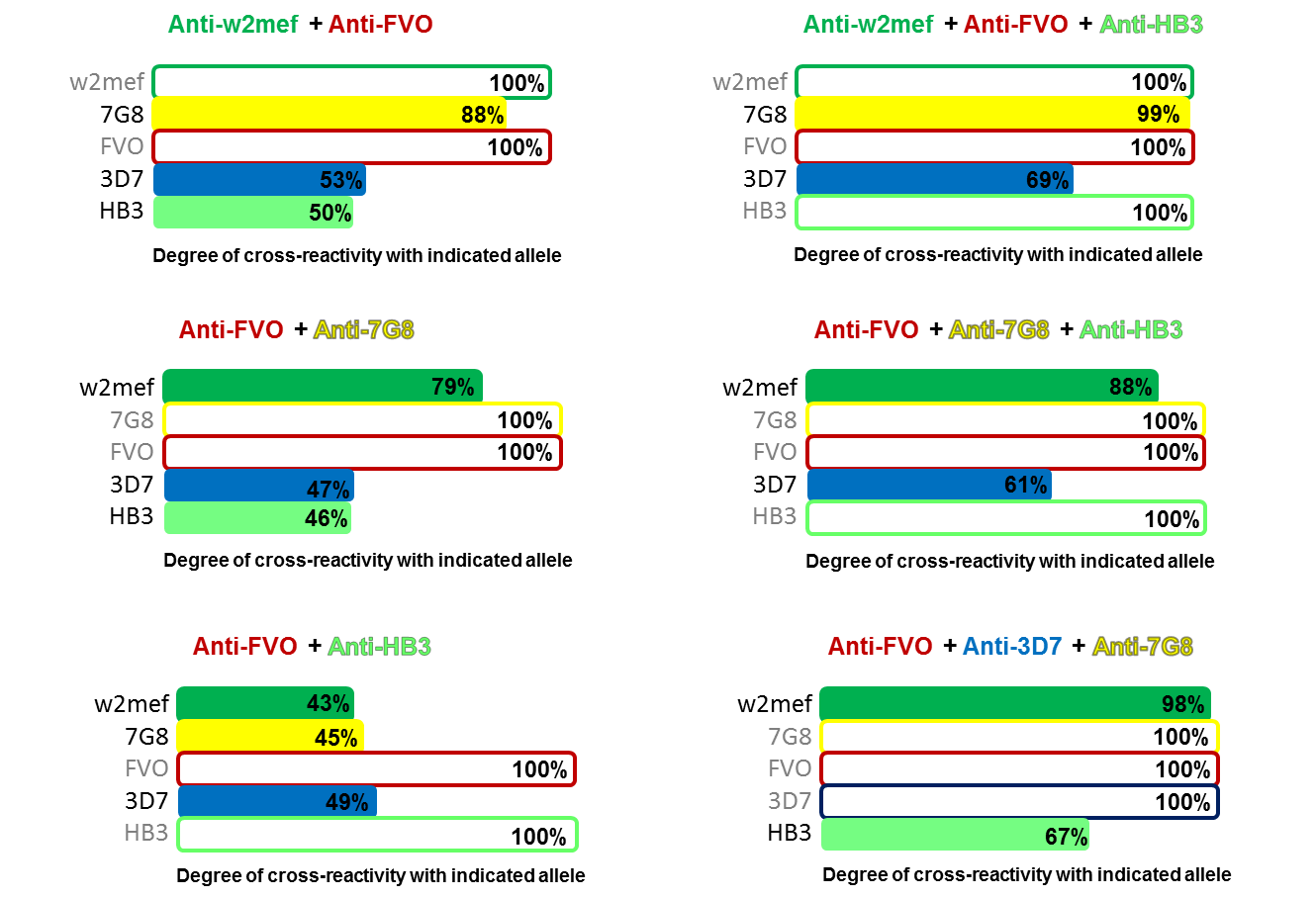


**G2**

**H2**

**I2**

**I1**

**H1**

**G1**

**J1**

**J2**


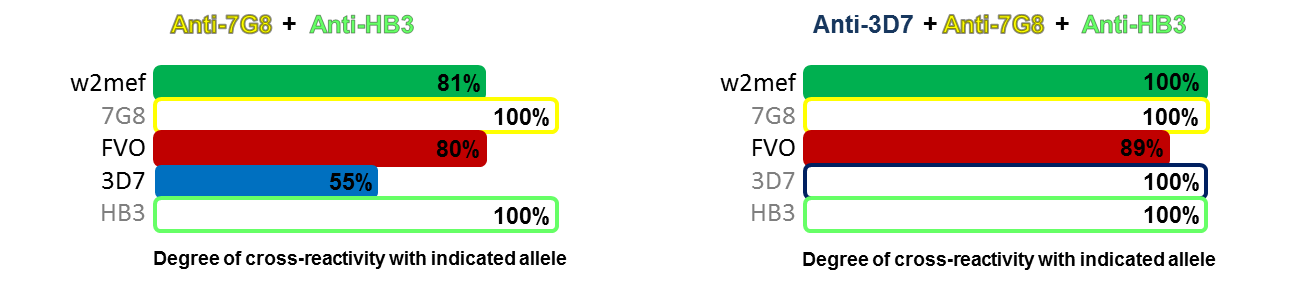


**Figure S7. AMA1 antibody cross-reactivity with multiple competitor alleles in competition ELISAs with samples from PNG children.**  Serum pools prepared from PNG children were tested for AMA1 antibody cross-reactivity in competition ELISAs using either two (A1-J1) or three (A2-J2) competitor AMA1 alleles. Enhancement of cross-reactivity by mixtures of two or three competitor alleles was dependent on the specific combination tested. The degree of cross-reactivity observed with a particular combination of competitor alleles indicates the extent to which immunisation with that combination might provide coverage against other alleles.

**A1**

**A2**

**
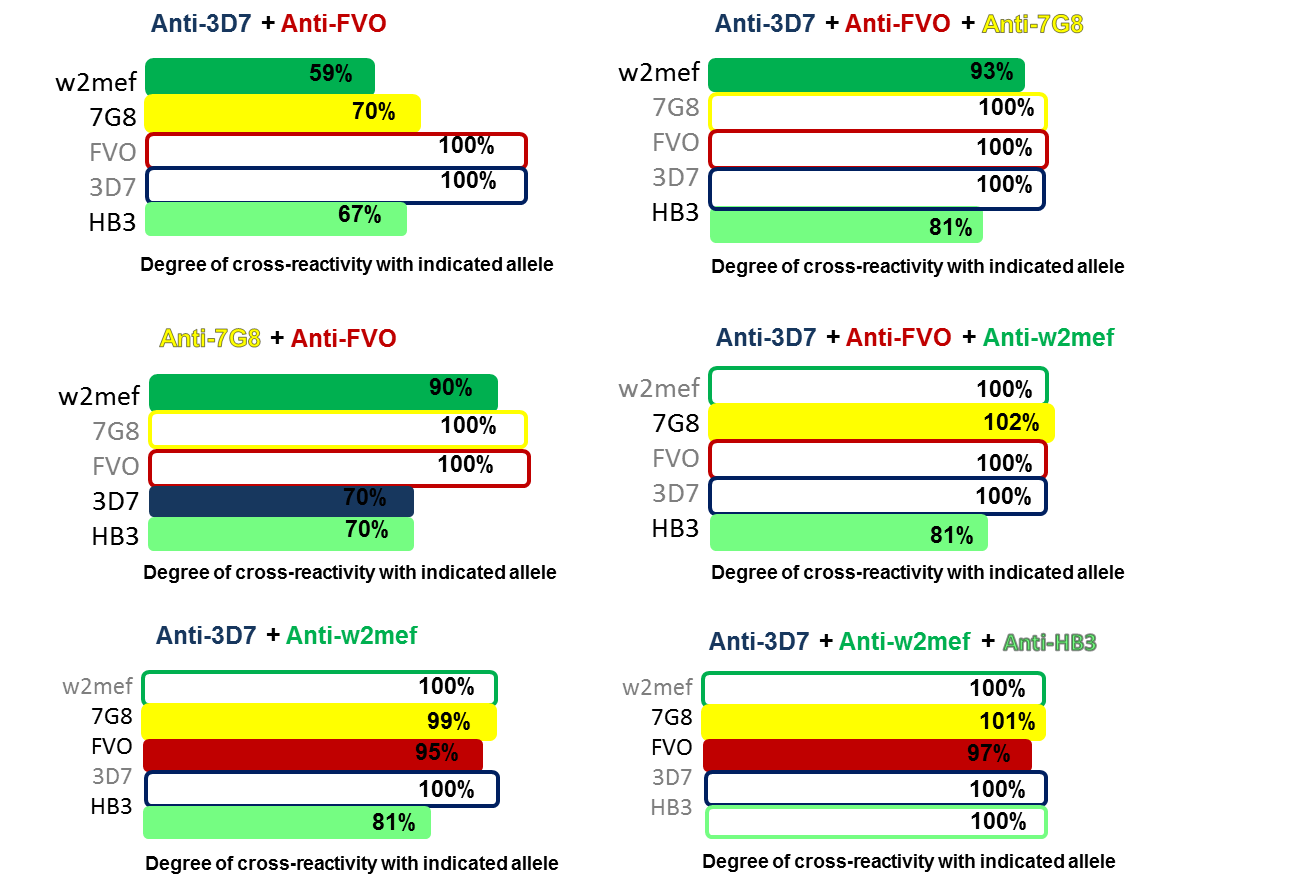
**

**C2**

**C1**

**B2**

**B1**

**Figure S8. AMA1 antibody cross-reactivity with multiple competitor alleles in competition ELISAs using samples from the Kenyan cohort.**  A pool of samples from the cohort of Kenyan children was tested for cross-reactivity of naturally acquired antibodies against 5 different PfAMA1 alleles. Selected combinations of two (A1-C1) or three (A2-C2) AMA1 alleles were tested in multiple antigen competition ELISA. As for the PNG cohort, cross-reactivity depended on the combination of competitor alleles.
